# Anti-Inflammatory Lobane and Prenyleudesmane Diterpenoids from the Soft Coral *Lobophytum varium*

**DOI:** 10.3390/md15100300

**Published:** 2017-09-29

**Authors:** Atallah F. Ahmed, Wan-Ting Teng, Chiung-Yao Huang, Chang-Feng Dai, Tsong-Long Hwang, Jyh-Horng Sheu

**Affiliations:** 1 Department of Marine Biotechnology and Resources, National Sun Yat-sen University, Kaohsiung 804, Taiwan; afahmed@ksu.edu.sa (A.F.A.); m025020024@student.nsysu.edu.tw (W.-T.T.); huangcy@mail.nsysu.edu.tw (C.-Y.H.); 2 Department of Pharmacognosy, College of Pharmacy, King Saud University, Riyadh 11451, Saudi Arabia; 3 Department of Pharmacognosy, Faculty of Pharmacy, Mansoura University, Mansoura 35516, Egypt; 4 Institute of Oceanography, National Taiwan University, Taipei 112, Taiwan; corallab@ntu.edu.tw; 5 Graduate Institute of Natural Products, College of Medicine, Chang Gung University, Taoyuan 333, Taiwan; htl@mail.cgu.edu.tw; 6 Research Center for Chinese Herbal Medicine, Research Center for Food and Cosmetic Safety, and Graduate Institute of Health Industry Technology, College of Human Ecology, Chang Gung University of Science and Technology, Taoyuan 333, Taiwan; 7 Department of Anesthesiology, Chang Gung Memorial Hospital, Taoyuan 333, Taiwan; 8 Institute of Natural Products, Kaohsiung Medical University, Kaohsiung 807, Taiwan; 9 Department of Medical Research, China Medical University Hospital, China Medical University, Taichung 404, Taiwan; 10 Frontier Center for Ocean Science and Technology, National Sun Yat-sen University, Kaohsiung 804, Taiwan; 11 Doctoral Degree Program in Marine Biotechnology, National Sun Yat-sen University, Kaohsiung 804, Taiwan

**Keywords:** soft coral, *Lobophytum varium*, lobane, prenyleudesmane, anti-inflammatory activity

## Abstract

New lobane-based diterpenoids lobovarols A–D (**1**–**4**) and a prenyleudesmane-type diterpenoid lobovarol E (**5**) along with seven known related diterpenoids (**6**–**12**) were isolated from the ethyl acetate extract of a Taiwanese soft coral *Lobophytum varium*. Their structures were identified on the basis of multiple spectroscopic analyses and spectral comparison. The absolute configuration at C-16 of the known compound **11** is reported herein for the first time. The anti-inflammatory activities of compounds **1**–**12** were assessed by measuring their inhibitory effect on *N*-formyl-methionyl-leucyl-phenyl-alanine/cytochalasin B (fMLP/CB)-induced superoxide anion generation and elastase release in human neutrophils. Metabolites **2**, **5**, and **11** were found to show moderate inhibitory activity on the generation of superoxide anion, while compounds **5**, **8**, **11**, and **12** could effectively suppress elastase release in fMLP/CB-stimulated human neutrophil cells at 10 μM. All of the isolated diterpenoids did not exhibit cytotoxic activity (IC_50_ > 50 μM) towards a limited panel of cancer cell lines.

## 1. Introduction

Marine organisms have been well recognized as an important source of natural products with diverse chemical structures and wide array of bioactivities, including the anti-inflammatory activities [1,2,3]. Soft corals belonging to the genus *Lobophytum* (Alcyoniidae) are considered to be a rich source of diterpenoids [4,5,6,7,8,9,10,11,12,13,14,15,16] and steroids [17,18,19,20,21,22], of which some exerted cytotoxic [4,5,6,7,8,18,21,23], antibacterial [4,8,10,11], antiviral [15], anti-acetylcholinesterase [9], and anti-inflammatory activities [6,8,10,15,16,19]. The terpenoid metabolites of cembrane-derived [4,5,6,7,8,9,15,16], including their dimers [8,10], lobane-derived diterpenoids [11,12,13,14], and to a lesser extent of prenyleudesmane-based diterpenoids [12,23,24], have been reported from soft corals belonging to the genus *Lobophytum*. Certain lobane diterpenoids exhibited an anti-inflammatory potential through different mechanisms such as reduction of cyclooxygenase-2 (COX-2) protein expression [25], inhibition of leukotriene synthesis [26], and suppression of 5-lipoxygenase (5-LOX) [27]. Our continuing investigation on the chemical constituents of soft corals belonging to the genus *Lobophytum* has led to the discovery of steroids, glycolipids, and cembranoids from *L. mirabile* [21], *L. crassum* [6,16,28], and *L. sarcophytoides* [19]. This study was aimed to isolate new metabolites from a Taiwanese soft coral *L. varium* and led to the discovery of five new diterpenes and seven known related compounds. Neutrophils play a critical role in the host defenses against invasion by pathogens and in the pathogenesis of various inflammatory diseases, such as rheumatoid arthritis. Activation of neutrophils by diverse stimuli results in secretion of a series of cytotoxins, reactive oxygen species (e.g., superoxide anion), and granule proteases (e.g., elastase) [29]. Therefore, agents which can suppress the excessive activation of neutrophils have been proposed to ameliorate the related inflammatory diseases. In this context, the anti-inflammatory activity of the isolated metabolites from *L. varium* was evaluated in vitro through measuring their ability to inhibit fMLP/CB-stimulated superoxide anion generation and elastase release in human neutrophils. This assay has been previously used by our group to disclose a number of marine diterpenoids possessing anti-inflammatory potential [30,31]. Moreover, the cytotoxicity against the growth of a limited panel of cancer cell lines was evaluated using Alamar Blue assay.

## 2. Results and Discussion

**Figure 1:**
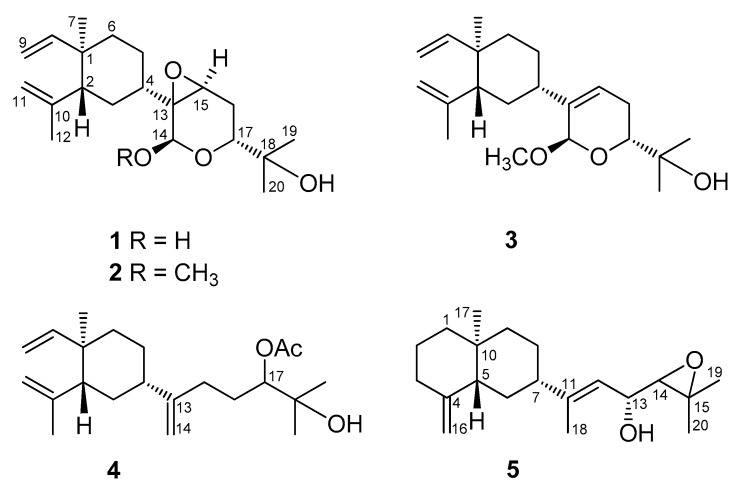
New diterpenoids isolated from *Lobophytum varium*.

**Figure 2:**
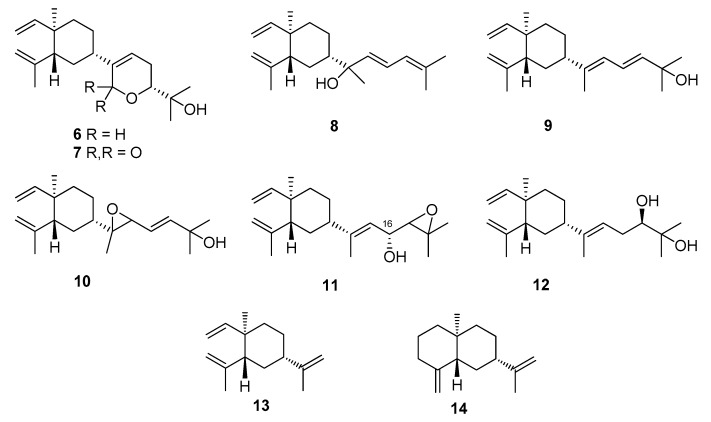
Known diterpenoids (**6**–**12**) isolated from *Lobophytum varium*, β-elemene, and β-silenene.

The solvent-free EtOAc extract of *L. varium* was primarily fractionated over a silica gel column. Further separation using a series of normal phase (NP) and reversed phase (RP) silica yielded five new diterpenoids lobovarols A–E (**1**–**5**, Figure 1) and seven known lobane diterpenoids (**6**–**12**, Figure 2). The chemical identities of the known compounds (**6**–**12**) were determined by comparison of their infrared (IR), mass spectrum (MS), and nuclear magnetic resonance (NMR) spectroscopic data with the published data and were found to be lobatriene (**6**) [32,33], lobatrienolide (**7**) [34], isofuscol (**8**) [24], fuscol (**9**) [35], 13,15-epoxyloba-8,10,16-trien-18-ol (**10**) [36], 17,18-epoxyloba-8,10,13(15)-trien-16-ol (**11**) [13,14], and (1*R*,2*R*,4*S*,17*R*)-loba-8,10,13(15)-trien-17,18-diol (**12**) [12], respectively.

**Figure 3:**
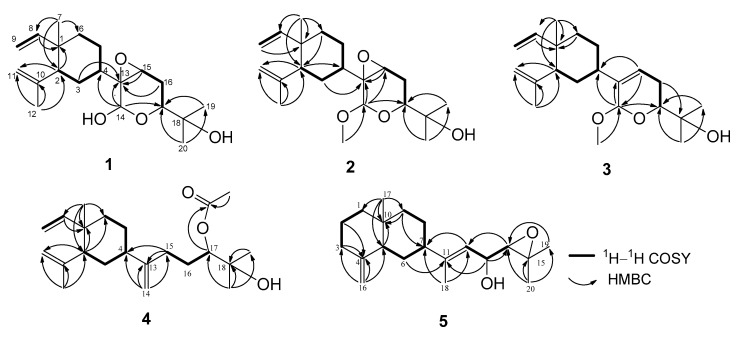
Key COSY and HMBC correlations of **1**–**5**.

Lobovarol A (**1**) was isolated as a colorless oil, [α]${}_{D}^{25}$ −31.7. The high-resolution electrospray ionization mass spectrometry (HRESIMS) (*m/z* 359.2191 [M + Na]^+^) and NMR data of **1** (Table 1 and Table 2) established the molecular formula of **1** as C_20_H_32_O_4_ with five degrees of unsaturation. The broad IR absorption band at ν_max_ 3417 cm^−1^ was ascribed to hydroxy functionality. The ^13^C NMR spectral data, measured in CDCl_3_ (Table 2) displayed twenty carbon signals, including those of four methyls, of a diterpenoid. The ^13^C and ^1^H NMR spectra of **1** revealed the presence of two olefins: a vinyl (δ_C_ 149.6, CH and 110.2, CH_2_; δ_H_ 5.78, dd, *J* = 17.6, 10.4 Hz, 4.90 d, *J* = 17.6 Hz, and 4.90, br d, *J* =10.4 Hz) and an isopropenyl (δ_C_ 147.1, C, 112.5, CH_2_ and 24.9, CH_3_; δ_H_ 4.84 and 4.60, each 1H, br s; and 1.70, 3H, s). Furthermore, a ring-junctured methyl (δ_C_ 16.5, CH_3_; δH 0.99, 3H, s) and a methine (δ_C_ 52.1, CH; δ_H_ 1.94, br dd, *J* = 9.2, 6.0 Hz) groups exhibited *^3^J_CH_* heteronuclear multiple bond correlations (HMBC) to each other and designated a β-elemene (**13**) ring system [12,13,37] in the molecule. These NMR signals are also characteristic for the lobane-type diterpenoids [11,12,13,14,25]. The presence of a trisubstituted epoxide (δ_C_ 64.2, C, and 59.1, CH; δ_H_ 3.49, dd, *J* = 2.0, 2.0 Hz), a dimethyl hydroxymethine (δ_C_ 71.1, C, 26.5, CH_3_, and 24.0, CH_3_; δ_H_ 1.22 and 1.13, each 3H s), an oxymethine (δ_C_ 68.3, CH; δ_H_ 3.45, dd, *J* = 11.2, 3.0 Hz) and a dioxy-methine (δ_C_ 89.5, CH; δ_H_ 5.30, s) were also confirmed in the side chain of the six-membered ring. Thus, an oxygen atom should form an ether-linkage at C-14 (δ_C_ 89.5, CH) and C-17 (δ_C_ 68.3, CH), which was confirmed by the HMBC correlations from H-14 (δ_H_ 5.30, s) to C-17. Comparison of ^13^C NMR spectral data of **1** with those of lobatrienolide (**7**) isolated from *Sinularia flexibilis* [34] and in this study, revealed the same carbon skeleton for both compounds. However, the carbonyl at C-14 (δ_C_ 164.7, C) and the trisubstituted double bond (δ_C_ 136.7, C, C-13 and 137.1, CH, C-15) of **7** have been reduced to a hemiketal methine group (δ_C_ 89.5, CH, C-14) and epoxidized (δ_C_ 64.2, C, C-13 and 59.1, CH, C-15) in **1**, respectively. Analysis of correlation spectroscopy (COSY) correlations of **1** established three consecutive proton spin systems extending from H-2 to H_2_-6, H-8 to H_2_-9, and H-15 to H-17 (Figure 3), which were connected by the key HMBC correlations observed from the angular methyl protons H_3_-7 (δ_H_ 0.99, 3H, s) to C-2, C-6, and C-8, and from the olefinic methyl protons H_3_-12 (δ_H_ 1.70, 3H, s) to C-2, and confirmed the β-elemene ring system. Moreover, HMBC correlations found from the hemiketal methine proton H-14 (δ_H_ 5.30, s) to C-4, C-13, and C-17, from each of H_3_-19 and H_3_-20 (δ_H_ 1.13 and 1.22, each 3H, s) to the oxymethine carbon C-17 confirmed the ether linkage of the 2-hydroxypyran ring and the epoxide ring to be at C-14/C-17 and C-13/C-15, respectively. Thus, the planar structures of **1** was established as shown in Figure 3.

**Figure 4:**
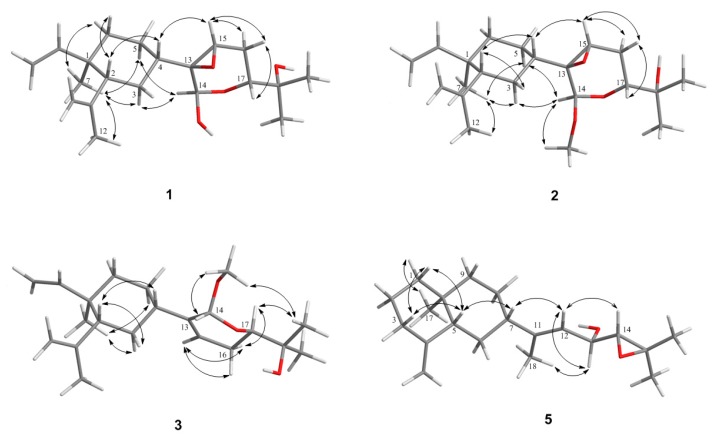
Key NOE correlations of **1**–**3** and **5**.

**Figure 5:**
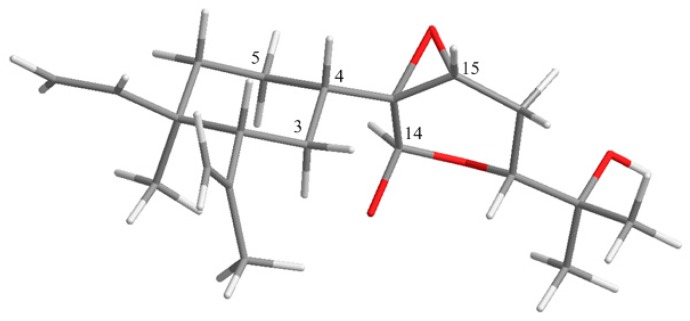
Molecular model of **1a**.

The relative configuration at the seven chiral centers of **1** was determined by the analysis of nuclear Overhauser effect (NOE) correlations along with molecular modeling using MM2 force field calculations (Figure 4). The nuclear Overhauser effect spectroscopy (NOESY) spectrum of compound **1** was remeasured in C_6_D_6_ for better resolution since the proton signals of H_2_-3 and H-4 in CDCl_3_ were overlapped (3H, 1.56, m). In C_6_D_6_, NOE interactions were observed for H-4 (δ_H_ 1.25, m) with H-2 (δ_H_ 1.75, m), H_3_-7 (δ_H_ 0.92, 3H, s) with H-6α (δ_H_ 1.22, m), H-6β (δ_H_ 1.33, m) with H-2. Furthermore, the similar δ_C_ values of C-1, C-2, C-7, and C-8 to C-12 of the previously-reported β-elemene and lobane-type diterpenoids [12,32,33,34], isolated from the same genus *Lobophytum* or prepared by enantiocontrolled synthesis [37], suggested the 1*R*,2*R*,4*S*-configuration in compound **1**. Moreover, the *R* configuration established for C-16 in the related lobane diterpenoids **11** (latter discussed) also implied the absolute configuration of chiral centers of the prenyleudesmane **5** and hence the lobanes **1**–**4**. The NOE correlations observed for both H-3α (δ_H_ 1.50, m) and H-5α (δ_H_ 1.22, m) with H-14 (δ_H_ 5.22, s), and for H-4 with H-15 (δ_H_ 3.03, dd, *J* = 2.0, 1.6 Hz) indicated that the protons at C-14 and C-15 of the pyran ring should be *syn* to each other and were assigned arbitrarily as α-oriented. In turn, H-15 exhibited NOE interactions with both H_2_-16 protons (δ_H_ 1.58, ddd, *J* = 13.2, 11.2, 2.0 Hz, H-16α and 1.73, m, H-16β) while H-17, which has an axial-axial coupling with H-16α (*J* = 11.2 Hz), displayed a significant NOE correlation with H-16β. Therefore, H-17 should be β-configured. This was also suggested by the absence of NOE response of H-17 with H-14. The above-mentioned NOEs found for H-14 with H-3α and H-5α, and for H-15 with H-4 revealed that the pyran ring should be perpendicular to the β-elemene ring system. To further prove the β-position of the epoxide ring, a conformation analysis using Chem3D, molecular mechanics calculations (MM2) and dihedral driving calculation were carried out [38,39]. The most stable (the lowest-energy) conformations for compound **1** and its 13,15-epimer **1a** which possesses an α-epoxide are represented in Figure 4 and Figure 5, respectively. In this perspective, we focused on the calculated distances between the diagnostic proton pairs having key NOE correlations in conformer **1**, which were found shorter than 3.0 Å, in comparison with those calculated for **1a** (Table 3). The results demonstrated that the β-configuration of the epoxide ring could only fulfill all described NOE correlations mentioned above. On the basis of the above findings, the (1*R*, 2*R*, 4*S*, 13*R*, 14*R*, 15*S*, 17*R*)-configuration of **1** was, thus, established.

Lobovarol B (**2**) was also isolated as a colorless oil with a hydroxy group (IR ν_max_ 3445 cm^−1^). The NMR data (Table 1 and Table 2) showed the characteristic signals of lobane-type diterpenoids as in **1**. Its HRESIMS *m*/*z* 373.2350 [M + Na]^+^ and NMR data deduced a molecular formula C_21_H_34_O_4_ with a 14 mass unit difference from compound **1**. Comparison of NMR data of compounds **2** and **1** revealed that compound **2** is the methyl ether of **1** due to the appearance of the methoxy signals (δ_C_ 55.6, CH_3_; δ_H_ 3.47, 3H, s). The HMBC correlation observed from the methoxy protons to the dioxymethine carbon (δ_C_ 97.7, CH, C-14) designated the C-14 position of the methoxyl. Therefore, compound **2** was identified as the methyl acetal arising from methylation of 14-OH of **1**. The structure of **2** was further confirmed by the analysis of COSY and HMBC correlations (Figure 3). Moreover, compound **2** displayed analogous NOE correlations and possessed the same sign of optical rotation ([α]${}_{D}^{25}$ −34.7) as those of **1**, implying the same absolute configuration for both **1** and **2**.

Lobovarol C (**3**) was obtained as a colorless oil. Its sodium adduct ion peak [M + Na]^+^ at *m/z* 357.2400 in the HRESIMS revealed a molecular formula of C_21_H_34_O_3_ which has one oxygen atom less than that of **2**. The IR absorption band at ν_max_ of 3450 cm^−1^ again indicated the presence of a hydroxy functionality in the molecule. Again, careful inspection of the ^1^H and ^13^C NMR spectroscopic data (Table 1 and Table 2) of **3** showed resonances and coupling constants identical to those of the β-elemene ring system, as verified in compounds **1** and **2** and other known lobane-type diterpenoids. Comparison of the 21 carbon signals of **3** with those of **2** showed that the trisubstituted epoxy signals in **2** was replaced by those of a trisubstituted double bond (δ_C/_δ_H_ 140.6, C; 121.1, CH/5.73, br d, *J* = 6.0 Hz) in **3**. The planar structure of **3** was further established by analyzing its COSY and HMBC correlations (Figure 3). Compound **3** exhibited NOE interactions consistent with 1*R*, 2*R*, and 4*S* configurations of the β-elemene ring system as in **1** and **2**. Additionally, the oxymethine proton H-17 (δ_H_ 3.67 dd, *J* = 11.6, 3.6 Hz) was found to NOE interact with one of the isopropyl group at C-18 (δ_H_ 1.29, 3H, s, H_3_-20) which, in turn, showed NOE correlation with the C-14 methoxyl protons. Thus, H-17 and H-14 are *anti* to each other, as found in compounds **1** and **2** (Figure 4). Compound **3** was, thus, identified as (1*R*,2*R*,4*S*,14*R*,17*R*,13*Z*)-14,17-epoxy-14-methoxyloba-8,10,13(15)-trien-18-ol.

The new metabolite lobovarol D (**4**) was found to have a molecular formula C_22_H_36_O_3_ as deduced from its HRESIMS (*m/z* 371.2557 [M + H]^+^) and NMR data (Table 1 and Table 2), implying five degrees of unsaturation. The IR absorptions at 3450 and 1735 cm^−1^ further indicated the presence of both hydroxy and ester functionalities. The NMR data revealed that compound **4** is another lobane diterpenoid possessing a β-elemene ring system and a side chain with an acetoxy, an exomethylene, and a tertiary hydroxyl. Analysis of the COSY spectrum of **4** revealed three consecutive protons systems (H-8/H_2_-9, H-2 to H_2_-6, and H_2_-15 to H-17). The connectivities of these three partial structures, as well as the location of the acetoxy, exomethylene, and the tertiary hydroxy groups, were established by inspection of the *^2^J_CH_* and *^3^J_CH_* correlations found in the HMBC spectra (Figure 3). The *^2^J_CH_* and *^3^J_CH_* correlations observed from the tertiary H_3_-19 (δ_H_ 1.21, 3H, s) and H_3_-20 (δ_H_ 1.22, 3H, s) to the *sp^3^* non-protonated oxycarbon (δ_C_ 72.5, C, C-18) and the oxymethine carbon (δ_C_ 79.7, CH, C-17) positioned the hydroxy and acetoxy groups at C-18 and C-17, respectively. Further, the HMBC correlations found from the olefinic protons (δ_H_ 4.82, and 4.74, each 1H, s) to C-4 (δ_C_ 44.4, CH), C-13 (δ_C_ 153.6, C), and C-15 (δ_C_ 31.4, CH_2_) indicated the C-13 location of the exomethylene group. These results established the gross structure of **4** (Figure 3). Analysis of NOE correlations again determined the 1*R*,2*R*,4*S* configuration of **4**. However, the C-17 configuration remains unresolved, although according to the related biosynthetic pathway, **4** might possess the same 17R configuration as those of **1**–**3**.

Lobovarol E (**5**) was obtained as a white powder. The molecular formula was deduced to be C_20_H_32_O_2_ as indicated by the HRESIMS (*m*/*z* 327.2292 [M + Na]^+^) and NMR data (Table 1 and Table 2), implying five degrees of unsaturation. Its IR absorption band at 3422 cm^−1^ revealed the presence of a hydroxy functionality, which was further supported by the NMR signals at δ_C_ 67.9 and δ_H_ 4.25. The NMR data (Table 1 and Table 2) showed the presence of one 1,1-disubstitued (δ_C_ 150.9, C and 105.4 CH_2_; δ_H_ 4.72 and 4.43, each 1H, s) and a trisubstituted (δ_C_ 146.1, C and 120.8, CH; δ_H_ 5.33, 1H, d, *J* = 8.5 Hz) olefinic bonds, a trisubstituted epoxide (δ_C_ 59.8, C; 67.5, CH; δ_H_ 2.82, 1H, d, *J* = 8.0 Hz), and a hydroxyl-bearing methine (δ_C_ 67.9, CH; δ_H_ 4.25, 1H, dd, *J* = 8.5, 8.0 Hz). One olfeinic methyl (δ_H_ 1.72, 3H, s), and three tertiary methyls (δ_H_ 1.33, 1.32, and 0.73, each 3H, s), were also identified. Therefore, the compound was suggested to have a bicyclic structure to fulfill the five degrees of unsaturation. The bicyclic structure of **5** was found to be the same as that of one eudesmene from the nearly the same NMR data of positions 1 to 10, 16, and 17 of **5** with the corresponding sesquiterpene (**14**) [40]. From the COSY correlations of **5** (Figure 3), three partial structures consecutive proton systems extended from H_2_-1 to H_2_-3, H-5 to H_2_-9, and H-12 to H-14 were established. Analysis of HMBC correlations of **5** led to the establishment of its planar structure. It was also found that the key HMBC correlations observed from both H_3_-19 and H_3_-20 to the epoxide carbons C-14 (δ_C_ 67.5, CH) and C-15 (δ_C_ 59.8, C) and from the hydroxymethine H-13 (δ_H_ 4.25, dd, *J* = 8.5, 8.0 Hz) to C-11 (δ_C_ 146.1, C) and C-14 demonstrated the positions of the epoxide and the hydroxyl to be at C-14/C-15 and C-13, respectively. This was further proved by the matched chemical shifts of ^1^H and ^13^C atoms of the side chain of **5** with the correspondent atoms of the known compound 17,18-epoxyloba-8,10,13(15)-trien-16-ol (**11**) [13] which was also isolated in this study. Therefore, the prenyleudesmane molecular structure of **5** was established as illustrated in Figure 3.

**Figure 6:**
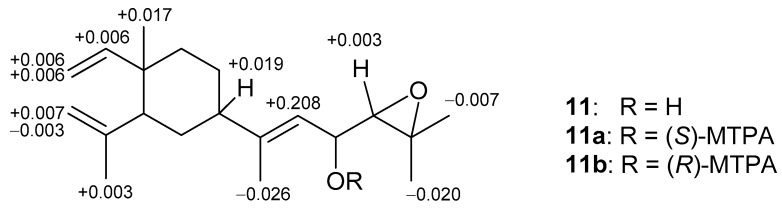
^1^H NMR chemical shift differences Δδ (δ_S_ − δ_R_) in ppm for the MTPA esters of **11**.

The relative configuration of **5** was determined by analyzing the NOE correlations in the NOESY spectrum, as well as a lowest energy stable conformation generated using MM2 calculation (Figure 4). The NOE interactions of H-5 with H-7, but not with H_3_-17, reflected the 5R*, 7S*, 10S*-configuration. The NOE correlations displayed for the β-oriented H-7 with the olefinic proton H-12, but not with H_3_-18, disclosed the *E* geometry of the 11,12-double bond. The α-orientation of the hydroxyl at C-13 was suggested by the NOE correlations of H-12/H-13 and H-12/H-7, as shown in a molecular model in Figure 4. The NOE correlations of H-12/H-7 and H_3_-18/H-13 proved the *E*-geometry of C-11/C-12 double bond. The above finding and other detailed NOE correlations (Figure 4) established the relative stereochemistry of **5**. The relative configuration at chiral carbons C-13 was further suggested by that correspondent to C-16 of the known biogenetically related metabolite **11** which has been also isolated from the same organism in this study. Fortunately, the larger quantity of compound **11** enabled us to determine the absolute configuration of **11** and hence that of **5**, through the esterification of 16-hydroxy group in **11** by Mosher’s method [41,42]. Analysis of the calculated Δδ_H_ (δ_S_ − δ_R_) values of protons neighboring C-16 of the prepared (*S*)- and (*R*)-2-methoxy-2-(trifluoromethyl)-2-phenylacetic (MTPA) esters (**11a** and **11b**, Figure 6) led to the assignment of the *R* configuration at C-16 in **11** and consequently the correspondent 13*R* configuration in **5**. On the basis of the above findings, the absolute configuration of **5** was established as 5*R*, 7*S*, 10*S*, 13*R.* However, the stereochemistry at C-14 remained undetermined in spite of the NOE correlation of H-14/H-12.

The cytotoxic activity of the isolated compounds (**1**–**12**) were screened against human lung adenocarcinoma (A549), human prostatic carcinoma (LN-cap), and human colon adenocarcinoma (DLD-1) cell lines using the Alamar Blue assay. The results showed that these compounds are not cytotoxic toward the three cancer cell lines.

**Figure 7:**
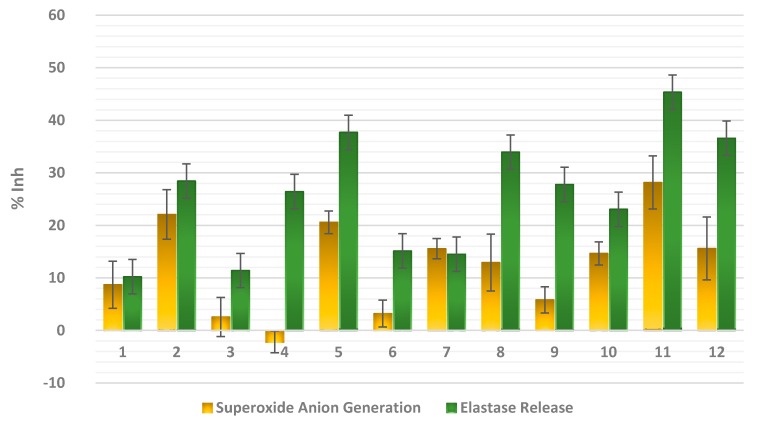
Inhibitory effects (% Inh) of compounds **1**–**12** at 10 μM on superoxide anion generation and elastase release by human neutrophils in response to *N*-formyl-l-methionyl-leucyl-phenylalanine/cytochalasin B (FMLP/CB). Results are presented as the mean ± S.E.M (*n* = 3–4).

Since many lobane diterpenoids were reported to exhibit anti-inflammatory activity through different mechanisms [25,26,27], the isolated metabolites in this study were evaluated for their anti-inflammatory potential through measuring their ability to suppress fMLP/CB-induced superoxide anion generation and elastase release in human neutrophils. The results (Figure 7) demonstrated that compounds **2**, **5**, and **11** expressed a moderate inhibitory effect (22.08 ± 4.71, 20.59 ± 2.15, and 28.16 ± 5.06%, respectively) at 10 μM against superoxide anion generation in fMLP/CB-stimulated cells. Moreover, compounds **5**, **8**, **11**, and **12** were found to be more active in inhibiting the elastase release (33.94 ± 5.85 to 45.34 ± 4.08%) than compounds **2**, **4**, **9**, and **10** which exhibited a moderate activity (23.07 ± 6.55 to 28.44 ± 5.28%) at 10 μM. The weak inhibition against elastase release was exerted by compounds **3**, **6**, and **7** (11.40 ± 1.28 to 15.14 ± 2.52%). It is noteworthy to mention that although compounds **5** and **11** possessed the same side chain, it seems that the ring system of β-elemene in **11** has a role in increasing the anti-inflammatory effect relative to β-selinene moiety. Moreover, except for compound **2**, other lobane diterpenoids possessing a pyran ring in their side chain (**1**, **3**, **6**, and **7**) showed weaker activity against elastase release in the fMLP/CB-stimulated neutrophils.

## 3. Materials and Methods

### 3.1. General Procedures

Optical rotations were measured on a JASCO P-1020 polarimeter (Jasco Corporation, Tokyo, Japan). IR spectra were recorded on a JASCO FT/IR-4100 spectrophotometer (Jasco). ESIMS and HRESIMS data were performed on a BRUKER APEX II mass (Bruker, Bremen, Germany) spectrometers. The NMR spectra were recorded on a Varian Unity INOVA 500 FT-NMR (Varian Inc., Palo Alto, CA, USA) at 500 MHz for ^1^H and 125 MHz for ^13^C or on a Varian 400 FT-NMR (Varian Inc.) at 400 MHz for ^1^H and 100 MHz for ^13^C in CDCl_3_ or C_6_D_6_ using TMS as internal standard (δ in ppm, *J* in Hz). Silica gel 60 (230–400 mesh, Merck, Darmstadt, Germany) pre-coated silica gel plates (Merck, Kieselgel 60 F254, 0.2 mm) were used for open CC and analytical TLC analysis, respectively. Isolation by HPLC was performed by a Hitachi L-2455 instrument (Hitachi Ltd., Tokyo, Japan) equipped with a reversed-phase (RP-18) column (ODS-3, 5 µm, 250 × 20 mm, Sciences Inc., Tokyo, Japan).

### 3.2. Animal Material

The soft coral *Lobophytum varium* Tixier-Durivault was collected by hand via SCUBA at a depth of 10–15 m from Jihui Fish Port, Taitung, Taiwan (23°7′2′′ N, 121°23′49.2′′ E), in March 2013, and stored at −20 °C until extraction. The organism was identified by Professor Chang-Feng Dai, Institute of Oceanography, National Taiwan University, Taipei 112, Taiwan. 

### 3.3. Extraction and Separation

The frozen bodies of *L. varium* (1.3 kg, wet weight) were sliced and exhaustively extracted with EtOAc. The solvent-free extract (55.4 g) was fractionated by silica gel column chromatography, using EtOAc in *n*-hexane (0.0 to 100%) then acetone in EtOAc (0.0 to 100%) as eluting solvents, to yield 24 fractions (F1 to F24). F8 eluted with 4.8% EtOAc in *n*-hexane was further purified in a silica gel column using EtOAc–*n*-hexane (1:10) to give two major subfractions F81 and F82. The subfractions were separately purified by NP-HPLC silica gel using 20% EtOAc in *n*-hexane and RP-HPLC silica gel using CH_3_CN-H_2_O (3:2) to yield **10** (1.8 mg) and **1** (2.8 mg), respectively. F9, eluted with 6.5% EtOAc in *n*-hexane, was initially purified in a silica gel column, using EtOAc–*n*-hexane (1:6), and then was isolated by RP-HPLC using MeOH to yield **8** (0.9 mg). F11, eluted with 9% EtOAc in *n*-hexane, was further fractionated successively in Sephadex LH-20 and RP-silica gel columns using MeOH and MeOH-H_2_O (3:1), respectively, to afford three subfractions, F111 to F113. The fractions were purified separately by RP-HPLC using MeOH-H_2_O (3:1, 4:1, and 5:1) to give **7** (1.8 mg), **2** (1.1 mg), and **6** (1.5 mg), respectively. F12, eluted with 11.5% EtOAc in *n*-hexane, was primarily purified by silica gel chromatography using EtOAc–*n*-hexane (1:10) and then separated by RP-HPLC using MeOH-H_2_O (8:1) to yield **3** (3.5 mg) and **9** (3.0 mg). Moreover, F15, eluted with 20% EtOAc in *n*-hexane, was re-chromatographed in a silica gel column using EtOAc–*n*-hexane (1:5), and then by RP-HPLC using MeOH-H_2_O (5:1) to afford **4** (2.8 mg). F18, eluted with 50% EtOAc in *n*-hexane, was initially refined in a silica gel column using EtOAc–*n*-hexane (1:5,) then further isolated on RP-HPLC using MeOH-H_2_O (5:1) to yield **11** (11 mg), **12** (6.1 mg), and **5** (2.6 mg).

#### 3.3.1. Lobovarol A (**1**)

Colorless oil; [α]${}_{D}^{25}$ −31.7 (*c* 0.70, CHCl_3_); IR (neat) ν_max_ 3417, 3081, 2925, 2856, 1639, 1561, 1377, 1033 cm^−1^; ^1^H NMR (400 MHz, CDCl_3_) and ^13^C (100 MHz, CDCl_3_) data, see Table 1 and Table 2, respectively; ^13^C (100 MHz, C_6_D_6_) δ_C_ 150.0 (CH, C-8), 147.2 (C, C-10), 112.8 (CH_2_, C-11), 110.3 (CH_2_, C-9), 89.9 (CH, C-14), 70.8 (C, C-18), 68.4 (CH, C-17), 64.2 (C, C-13), 58.8 (CH, C-15), 52.1 (CH, C-2), 41.9 (CH, C-4), 39.9 (C, C-1), 39.3 (CH_2_, C-6), 28.9 (CH_2_, C-3), 26.5 (CH_3_, C-20), 25.1 (CH_2_, C-16), 25.0 (CH_3_, C-12), 24.6 (CH_3_, C-19), 23.0 (CH_2_, C-5), 16.6 (CH_3_, C-7); ^1^H NMR (400 MHz, C_6_D_6_) δ_H_ 5.73 (1H, dd, *J* = 17.2, 10.8 Hz, H-8), 5.22 (1H, s, H-14), 4.92 (1H, d, *J* = 17.2 Hz, H-9β), 4.90 (1H, d, *J* = 10.8 Hz, H-9α), 4.89 (1H, s, H-11β), 4.66 (1H, s, H-11α), 3.37 (1H, dd, *J* = 11.2, 3.2 Hz, H-17), 3.03 (1H, dd, *J* = 2.0, 1.6 Hz, H-15), 1.75 (1H, m, H-2), 1.73 (1H, m, H-16β), 1.68 (3H, s, H_3_-12), 1.58 (1H, ddd, *J* = 13.2, 11.2, 2.0 Hz, H-16α), 1.53 (1H, m, H-3β), 1.50 (1H, m, H-3α), 1.48 (1H, m, H-5β), 1.33 (1H, m, H-6β), 1.25 (1H, m, H-4), 1.22 (2H, m, H-5α and H-6α), 1.19 (3H, s, H_3_-20), 1.05 (3H, s, H_3_-19), 0.92 (3H, s, H_3_-7); ESIMS *m/z* 359 [M + Na]^+^ and 375 [M + K]^+^; HRESIMS *m/z* 359.2191 [M + Na]^+^ (calcd. for C_20_H_32_O_4_Na, 359.2193).

#### 3.3.2. Lobovarol B (**2**)

Colorless oil; [α]${}_{D}^{25}$ −34.7 (*c* 0.28, CHCl_3_); IR (neat) ν_max_ 3445, 3079, 2925, 2857, 1641, 1539, 1374, 1048 cm^−1^; ^1^H NMR (500 MHz, CDCl_3_) and ^13^C (125 MHz, CDCl_3_) data, see Table 1 and Table 2, respectively; ESIMS *m/z* 373 [M + Na]^+^ and 389 [M + K]^+^; HRESIMS *m/z* 373.2350 [M + Na]^+^ (calcd. for C_21_H_34_O_4_Na, 373.2349).

#### 3.3.3. Lobovarol C (**3**)

Colorless oil; [α]${}_{D}^{25}$ −19.6 (*c* 0.88, CHCl_3_); IR (neat) ν_max_ 3450, 3080, 2927, 2865, 1639, 1459, 1374, 1045 cm^−1^; ^1^H NMR (400 MHz, CDCl_3_) and ^13^C (100 MHz, CDCl_3_) data, see Table 1 and Table 2, respectively; ESIMS *m/z* 357 [M + Na]^+^ and 373 [M + K]^+^; HRESIMS *m/z* 357.2400 [M + Na]^+^ (calcd. for C_21_H_34_O_4_Na, 357.2400).

#### 3.3.4. Lobovarol D (**4**)

Colorless oil; [α]${}_{D}^{25}$ +12.3 (*c* 0.70, CHCl_3_); IR (neat) ν_max_ 3450, 3080, 2924, 2857, 1735, 1641, 1458, 1459, 1373, 1242, 1041 cm^−1^; ^1^H NMR (400 MHz, CDCl_3_) and ^13^C (100 MHz, CDCl_3_) data, see Table 1 and Table 2, respectively; ESIMS *m/z* 371 [M + Na]^+^; HRESIMS *m/z* 371.2557 [M + Na]^+^ (calcd. for C_21_H_34_O_4_Na, 371.2557).

#### 3.3.5. Lobovarol E (**5**)

White amorphous powder; [α]${}_{D}^{25}$ −12.7 (*c* 0.65, CHCl_3_); IR (neat) ν_max_ 3422, 3080, 2927, 2864, 1648, 1453, 1380, 1245, 1057 cm^−1^; ^1^H NMR (500 MHz, CDCl_3_) and ^13^C (125 MHz, CDCl_3_) data, see Table 1 and Table 2, respectively; ESIMS *m/z* 327 [M + Na]^+^; HRESIMS *m/z* 327.2292 [M + Na]^+^ (calcd. for C_21_H_34_O_4_Na, 327.2295).

#### 3.3.6. Preparation of (*S*)- and (*R*)-MTPA Esters of **11**

To a solution of 11a (2.0 mg) in pyridine (100 μL), *R*-(−)-MTPA chloride (5 μL) was added and allowed to react overnight at RT. The reaction was terminated by the addition of 1.0 mL of water, and then processed as previously described [43] to yield the (*S*)-MTPA ester **11a** (0.4 mg, 19%). Similarly, the correspondent (*R*)-MTPA ester **11** was also obtained from the reaction of *S*-(+)-MTPA chloride with **11** to give **11b** (0.2, 11%). ^1^H NMR (CDCl_3_, 400 MHz) of **11a**: δ_H_ 5.819 (1H, dd, *J* = 18.0, 10.8 Hz, H-8), 5.631 (1H, dd, *J* = 10.0, 8.4 Hz, H-16), 5.3665 (1H, d, *J* = 10 Hz, H-15), 4.9225 (1H, dd, *J* = 18.0, 1.2 Hz, H-9b), 4.920 (1H, dd, *J* = 10.8, 1.2 Hz, H-9a), 4.849 (1H, dd, *J* = 2.0, 2.0 Hz, H-11b), 4.593 (1H, s, H-11a), 3.0005 (1H, dd, *J* = 8.4, 3.2 Hz, H-17), 2.0195 (1H, m, H-4), 1.8325 (3H, d, *J* = 1.2 Hz, H_3_-14), 1.7205 (3H, d, *J* = 0.4 Hz, H_3_-12), 1.343 (3H, s, H_3_-20), 1.328 (3H, s, H_3_-19), 1.020 (3H, s, H_3_-7); ^1^H NMR (CDCl_3_, 400 MHz) of **11b**: δ_H_ 5.813 (1H, dd, *J* = 18.0, 10.8 Hz, H-8), 5.597 (1H, dd, *J* = 10.0, 8.4 Hz, H-16), 5.1585 (1H, d, *J* = 10 Hz, H-15), 4.9165 (1H, dd, *J* = 18.0, 1.2 Hz, H-9b), 4.914 (1H, br d, *J* = 12.0 Hz, H-9a), 4.852 (1H, br s, H-11b), 4.586 (1H, s, H-11a), 2.9975 (1H, br d, *J* = 8.4 Hz, H-17), 2.0004 (1H, m, H-4), 1.8585 (3H, d, *J* = 1.2 Hz, H_3_-14), 1.718 (3H, s, H_3_-12), 1.363 (3H, s, H_3_-20), 1.335 (3H, s, H_3_-19), 1.003 (3H, s, H_3_-7).

### 3.4. Cytotoxicity Assay

Cancer cell (A549, LN-cap, and DLD-1) lines were purchased from the American Type Culture Collection (ATCC). Alamar Blue assay [44,45] protocol was used to evaluate the cytotoxicity for the isolated metabolites from *L. varium*.

### 3.5. In Vitro Anti-Inflammatory Assay

Human neutrophils were obtained from whole blood using dextran sedimentation and Ficoll centrifugation. Purified neutrophils were resuspended in a Ca^2+^-free HBSS buffer (pH 7.4) at 4 °C prior to use.

#### 3.5.1. Measurement of Superoxide Anion Generation

The production of superoxide anion was assayed by the method based on the superoxide oxide dismutase inhibitable reduction of ferricytochrome c [46,47]. Briefly, neutrophils incubated with ferricytochrome c (0.5 mg/mL) and Ca^2+^ (1 mM) were equilibrated at 37 °C for 2 min and then treated with different concentrations of the tested compounds for 5 min. Cells were activated by 100 nM fMLP for 10 min in the pretreatment of cytochalasin B (CB, 1 μg/mL) for 3 min (fMLP/CB).

#### 3.5.2. Measurement of Elastase Release

The elastase release was assayed using MeO-Suc-Ala-Ala-Pro-Val-*p*-nitroanilide as substrate [46]. Briefly, neutrophils incubated with MeO-Suc-Ala-Ala-Pro-Val-*p*-nitroanilide (100 μM) were equilibrated at 37 °C and then treated with the tested compounds for 5 min. Cells were then activated with fMLP (100 nM)/CB (0.5 μg/mL) for 10 min.

## 4. Conclusions

The ethyl acetate extract of a Taiwanese soft coral *Lobophytum varium* was chemically investigated for the first time and led to the discovery of four new lobane-based (**1**–**4**), and one new prenyleudesmane-type (**5**), diterpenoids, along with seven known related metabolites (**6**–**12**). The establishment of the absolute configuration of **11** was achieved by Mosher’s esterification. The evaluation of anti-inflammatory activity showed that diterpenoids **2**, **5**, and **11** possess moderate inhibitory activity on the generation of superoxide anion, while **5**, **8**, **11**, and **12** could effectively suppress elastase released after stimulation of human neutrophils by fMLP/CB. The active metabolites might be considered as promising leads in the development of anti-inflammatory drugs.

## Figures and Tables

**Table 1:** ^1^H NMR spectral data for compounds **1**–**5**.

#	1 ^a^	2 ^b^	3 ^a^	4 ^a^	5 ^b^
1	-	-	-	-	1.46 m; 1.28 m
2	1.94 br dd (9.2, 6.0) ^c^	1.95 dd (6.0, 6.0, 3.0)	2.02 dd (12.4, 4.0)	2.00 m	1.62, 2H, m
3	1.56, 2H, m	1.59 m; 1.49 m	1.61 m; 1.54 m	1.54, 2H, m	2.31 d (13.0); 2.01 m
4	1.56 m	1.56 m	2.00 m	1.89 m	
5	1.64 m; 1.36 m	1.63 m; 1.33 m	1.64 m; 1.49 m	1.61 m; 1.41 m	1.83 d (12.0)
6	1.38–1.50, 2H, m	1.46, 2H, m	1.48, 2H, m	1.46, 2H, m	1.54 m; 1.34 m
7	0.99, 3H, s	0.98 3H, s	1.00, 3H, s	1.00, 3H, s	1.97 m
8	5.78 dd (17.6, 10.4)	5.79 dd (18.0, 10.5)	5.82 dd (17.6, 10.4)	5.82 dd (18.0, 10.8)	1.53 m, 1.28 m
9	4.90 d (17.6); 4.90 d (10.4)	4.90 d (16); 4.89 d (13.5)	4.92 d (17.6); 4.91 d (10.4)	4.92 d (16.4); 4.90 d (11.6)	1.52, m; 1.28 m
11	4.84 br s; 4.60 br s	4.83 s; 4.58 s	4.82 dd (1.6, 1.6); 4.58 br d (1.6)	4.82 s; 4.59 s	-
12	1.70 3H, s	1.70 3H, s	1.71 3H, s	1.71, 3H, s	5.33 d (8.5)
13					4.25 dd (8.5, 8.0)
14	5.30 s	4.91 s	4.88 s	4.82 s; 4.74 s	2.82 d (8.0)
15	3.49 dd (2.0, 2.0)	3.30 br s	5.73 br d (6.0)	2.03, 2H, m	-
16	2.04 ddd (14.8, 2.4, 2.4); 1.82 ddd (14.8, 11.2, 2.0)	2.05 ddd (14.0, 2.5, 2.5); 1.84 ddd (14.0, 12.0, 2.5)	2.22 m; 1.99 m	1.75, 2H, m	4.72 s; 4.43 s
17	3.45 dd (11.2, 3.0)	3.58 dd (12, 2.5)	3.67 dd (11.6, 3.6)	4.84 dd (10.4, 2.8)	0.73, 3H, s
18	-	-	-	-	1.72, 3H, s
19	1.13, 3H, s	1.14, 3H, s	1.18, 3H, s	1.21, 3H, s	1.33, 3H, s
20	1.22, 3H, s	1.25, 3H, s	1.29, 3H, s	1.22, 3H, s	1.32, 3H, s
OMe	-	3.47, 3H, s	3.46 3H, s	-	-
OAc	-	-	-	2.13, 3H, s	-

Spectra recorded in CDCl_3_ at ^a^ 400 and ^b^ 500 MHz at 25 °C. ^c^ *J* values (Hz) in parentheses.

**Table 2:** ^13^C NMR data of compounds **1**–**5**.

#	1 ^a^	2 ^b^	3 ^a^	4 ^a^	5 ^b^
1	39.7 (C)	39.7 (C)	39.7 (C)	39.8 (C)	41.9 (CH_2_)
2	52.1 (CH) ^c^	52.0 (CH)	52.8 (CH)	52.8 (CH)	23.4 (CH_2_)
3	28.7 (CH_2_)	28.9 (CH_2_)	34.1 (CH_2_)	33.3 (CH_2_)	36.9 (CH_2_)
4	41.9 (CH)	41.8 (CH)	40.5 (CH)	44.4 (CH)	150.9 (C)
5	22.8 (CH_2_)	22.9 (CH_2_)	26.4 (CH_2_)	27.2 (CH_2_)	49.9(CH)
6	39.1 (CH_2_)	39.2 (CH_2_)	39.8 (CH_2_)	40.0 (CH_2_)	29.2 (CH_2_)
7	16.5 (CH_3_)	16.5 (CH_3_)	16.6 (CH_3_)	16.6 (CH_3_)	47.6 (CH)
8	149.6 (CH)	149.8 (CH)	150.2 (CH)	150.2 (CH)	26.6 (CH_2_)
9	110.2 (CH_2_)	110.1 (CH_2_)	109.9 (CH_2_)	109.9 (CH_2_)	41.0 (CH_2_)
10	147.1 (C)	147.2 (C)	147.5 (C)	147.6 (C)	36.0 (C)
11	112.5 (CH_2_)	112.4 (CH_2_)	112.2 (CH_2_)	112.1 (CH_2_)	146.1 (C)
12	24.9 (CH_3_)	24.7 (CH_3_)	24.7 (CH_3_)	24.8 (CH_3_)	120.8 (CH)
13	64.2 (C)	61.7 (C)	140.6 (C)	153.6 (C)	67.9 (CH)
14	89.5 (CH)	97.7 (CH)	97.8 (CH)	107.5 (CH_2_)	67.5 (CH)
15	59.1 (CH)	55.3 (CH)	121.1 (CH)	31.4 (CH_2_)	59.8 (C)
16	24.8 (CH_2_)	25.2 (CH_2_)	24.8 (CH_2_)	28.1 (CH_2_)	105.4 (CH_2_)
17	68.3 (CH)	69.5 (CH)	72.1 (CH)	79.7 (CH)	16.4 (CH)
18	71.1 (C)	71.4 (C)	71.5 (C)	72.5 (C)	15.5 (CH_3_)
19	24.0 (CH_3_)	24.5 (CH_3_)	24.4 (CH_3_)	24.9 (CH_3_)	24.9 (CH_3_)
20	26.5 (CH_3_)	27.0 (CH_3_)	26.7 (CH_3_)	26.8 (CH_3_)	19.6 (CH_3_)
14-OMe		55.6 (CH_3_)	55.4 (CH_3_)		
17-OAc				21.1 (CH_3_)	
				171.3 (C)	

Spectra recorded in CDCl_3_ at ^a^ 100 and ^b^ 125 MHz at 25 °C. ^c^ Attached protons were determined by DEPT experiments. Values are presented as ppm downfield from TMS.

**Table 3:** Calculated conformational energies as a function of the dihedral angle of C(3)-C(4)-C(13)-O and the distances between the diagnostic protons of **1** and **1a**.

Compound	1 (β-Epoxide)	1a (α-Epoxide)
Dihedral angle of C(3)-C(4)-C(13)-O	−80°	−150°
Minimum energy conformer (Kcal/mol)	75.78	80.92
Calculated distances		
H(4)-H(15)	2.45 Å	2.45 Å
H(14)-H(3α)	2.54 Å	3.73 Å
H(14)-H(5α)	2.30 Å	2.51 Å
H(14)-H(5β)	3.35 Å	2.79 Å

## Supplementary Materials

HRESIMS, ^1^H, and ^13^C spectra of new compounds **1**–**5** are available online at www.mdpi.com/1660-3397/15/10/300/s1. **Figure S1.** HRESIMS spectrum of **1**; **Figure S2.** ^1^H NMR spectrum of **1** in C_6_D_6_ at 400 MHz; **Figure S3.** ^13^C NMR spectrum of **1** in C_6_D_6_ at 100 MHz; **Figure S4.** HRESIMS spectrum of **2**; **Figure S5.** ^1^H NMR spectrum of **2** in CDCl_3_ at 500 MHz; **Figure S6.** ^13^C NMR spectrum of **2** in CDCl_3_ at 125 MHz; **Figure S7.** HRESIMS spectrum of **3**; **Figure S8.** ^1^H NMR spectrum of **3** in CDCl_3_ at 400 MHz; **Figure S9.** ^13^C NMR spectrum of **3** in CDCl_3_ at 100 MHz; **Figure S10.** HRESIMS spectrum of **4**; **Figure S11.** ^1^H NMR spectrum of **4** in CDCl_3_ at 400 MHz; **Figure S12.** ^13^C NMR spectrum of **4** in CDCl_3_ at 100 MHz; **Figure S13.** HRESIMS spectrum of **5**; **Figure S14.** ^1^H NMR spectrum of **5** in CDCl_3_ at 500 MHz; **Figure S15.** ^13^C NMR spectrum of **5** in CDCl_3_ at 125 MHz.
