# Supplementary material for: Anti-Inflammatory Lobane and Prenyleudesmane Diterpenoids from the Soft Coral Lobophytum varium
Source: Mar Drugs. 2017 Sep 29;15(10):300. doi: 10.3390/md15100300 (PMC5666408; doi:10.3390/md15100300)
Supplement: Supplementary file 1 [file marinedrugs-15-00300-s001.pdf]

Supplementary Materials: Anti-inflammaory Lobane and Prenyleudesmane Diterpenoids from the Soft Coral *Lobophytum varium*

Atallah F. Ahmed, Wan-Ting Teng, Chiung-Yao Huang, Chang-Feng Dai, Tsong-Long Hwang and Jyh-Horng Sheu

List of Supplementary material

No.	Content	Page
**Figure S1**	HRESIMS spectrum of **1**	S2
**Figure S2**	^{1}H NMR spectrum of **1** in C_6D_6 at 400 MHz	S3
**Figure S3**	^{13}C NMR spectrum of **1** in C_6D_6 at 100 MHz	S4
**Figure S4**	HRESIMS spectrum of **2**	S5
**Figure S5**	^{1}H NMR spectrum of **2** in $CDCl_3$ at 500 MHz	S6
**Figure S6**	^{13}C NMR spectrum of **2** in $CDCl_3$ at 125 MHz	S7
**Figure S7**	HRESIMS spectrum of **3**	S8
**Figure S8**	^{1}H NMR spectrum of **3** in $CDCl_3$ at 400 MHz	S9
**Figure S9**	^{13}C NMR spectrum of **3** in $CDCl_3$ at 100 MHz	S10
**Figure S10**	HRESIMS spectrum of **4**	S11
**Figure S11**	^{1}H NMR spectrum of **4** in $CDCl_3$ at 400 MHz	S12
**Figure S12**	^{13}C NMR spectrum of **4** in $CDCl_3$ at 400 MHz	S13
**Figure S13**	HRESIMS spectrum of **5**	S14
**Figure S14**	^{1}H NMR spectrum of **5** in $CDCl_3$ at 500 MHz	S15
**Figure S15**	^{13}C NMR spectrum of **5** in $CDCl_3$ at 125 MHz	S16

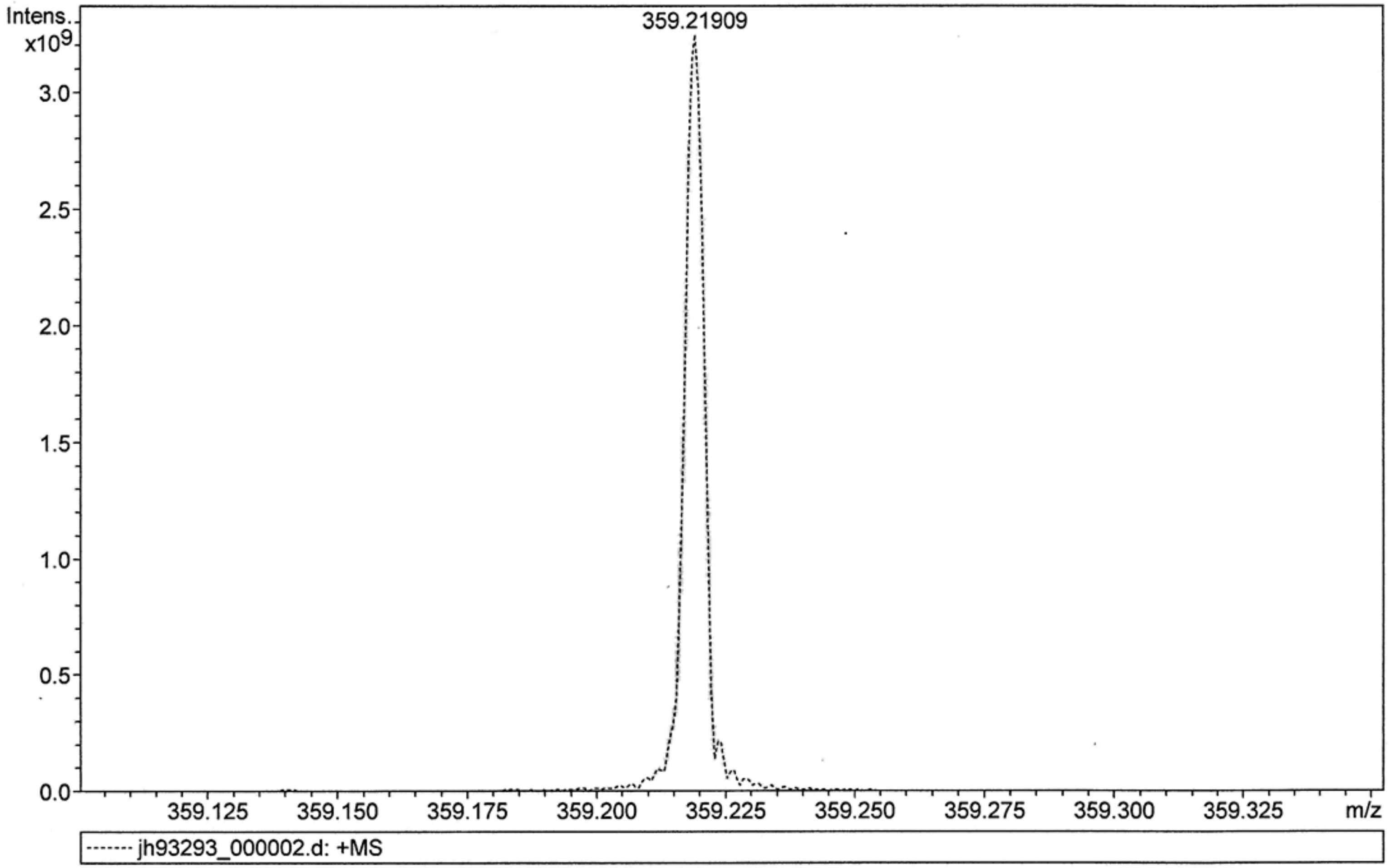

Meas. m/z	#	Formula	Score	m/z	err [mDa]	err [ppm]	mSigma	rdb	e⁻ Conf	N-Rule
359.21909	1	C 20 H 32 Na O 4	100.00	359.21928	0.19	0.54	9.8	4.5	even	ok

Figure S1. HRESIMS spectrum of **1**

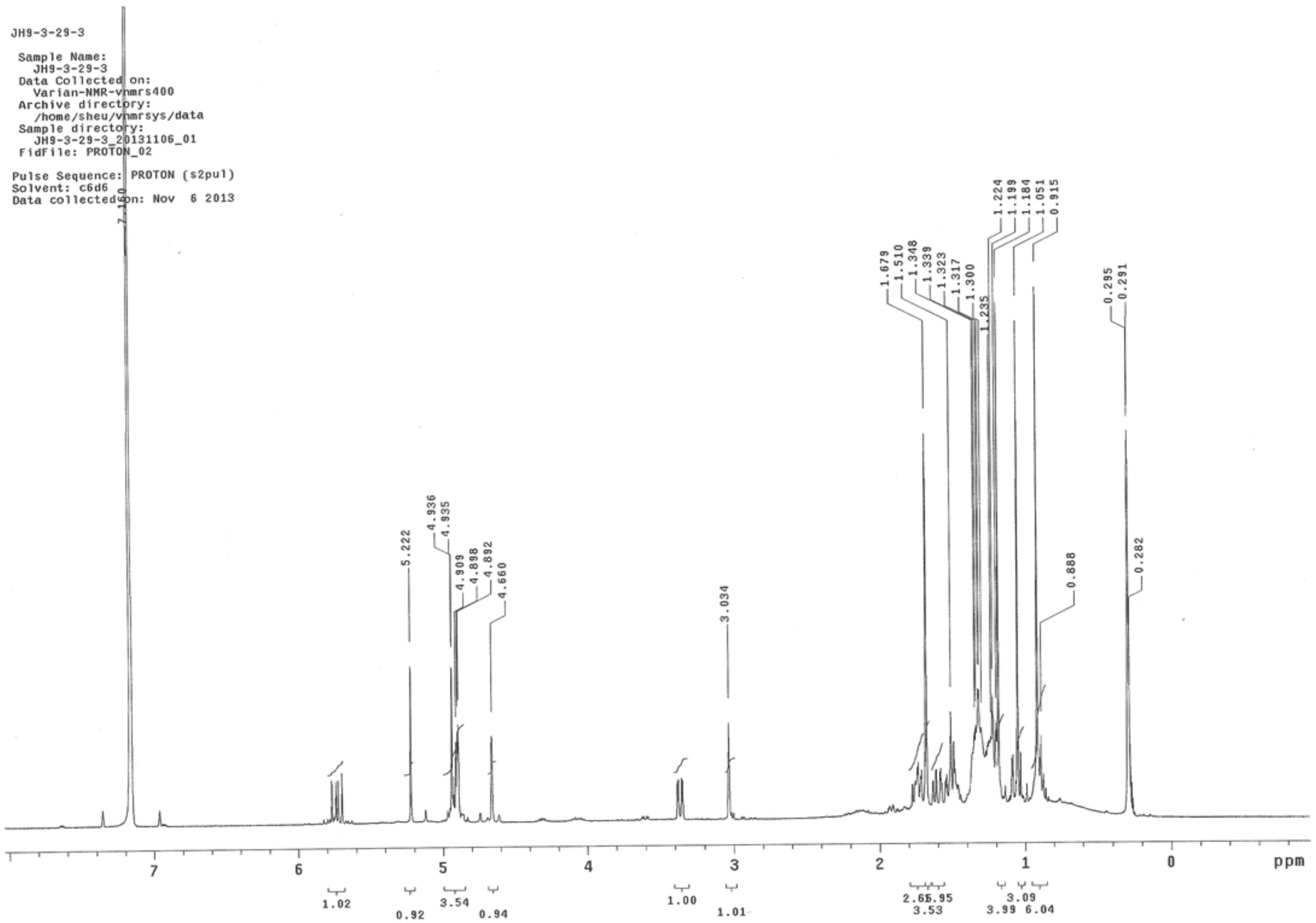

Figure S2. ^{1}H NMR spectrum of **1** in C_6D_6 at 400 MHz

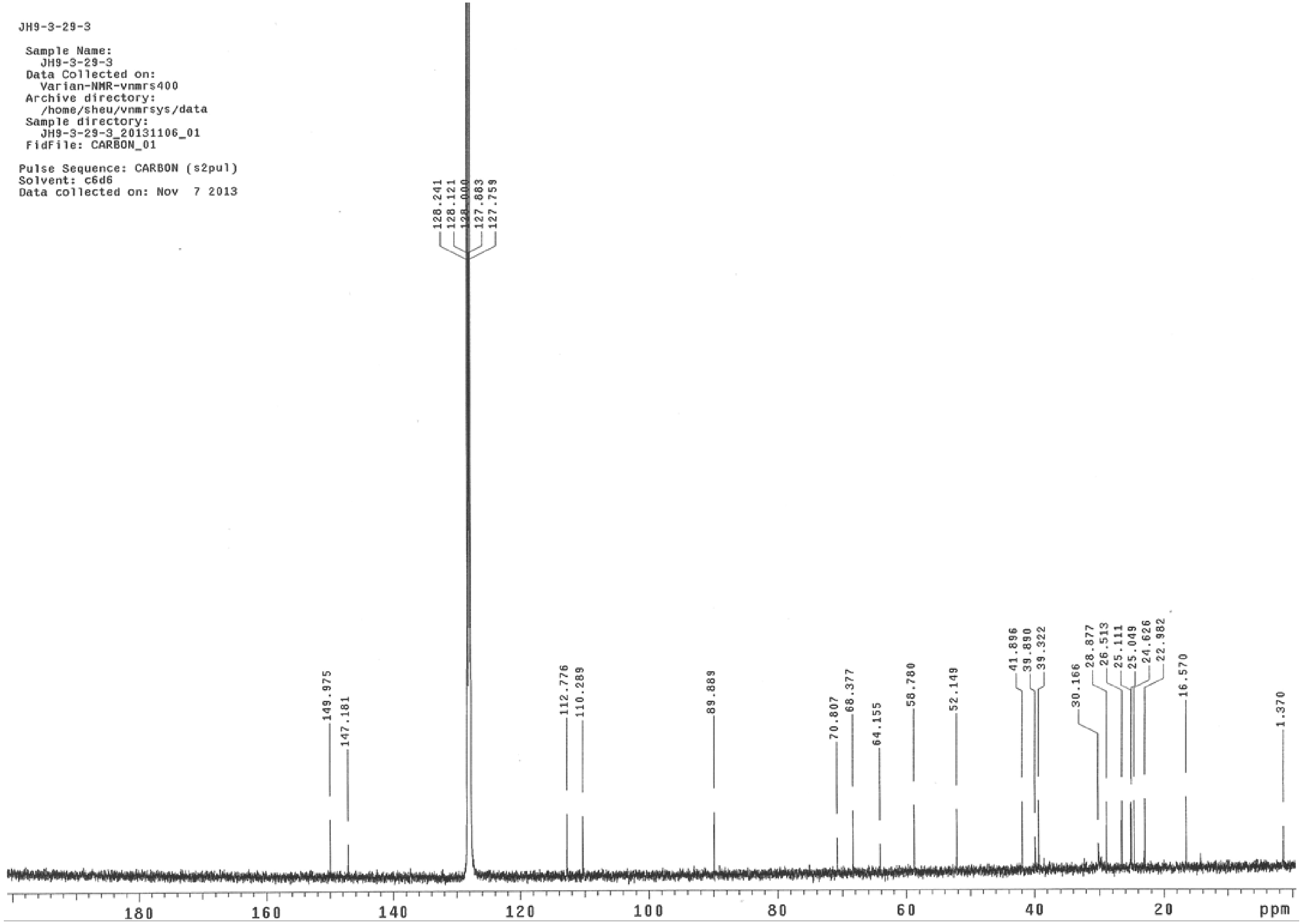

Figure S3. ^{13}C NMR spectrum of **1** in C_6D_6 at 100 MHz

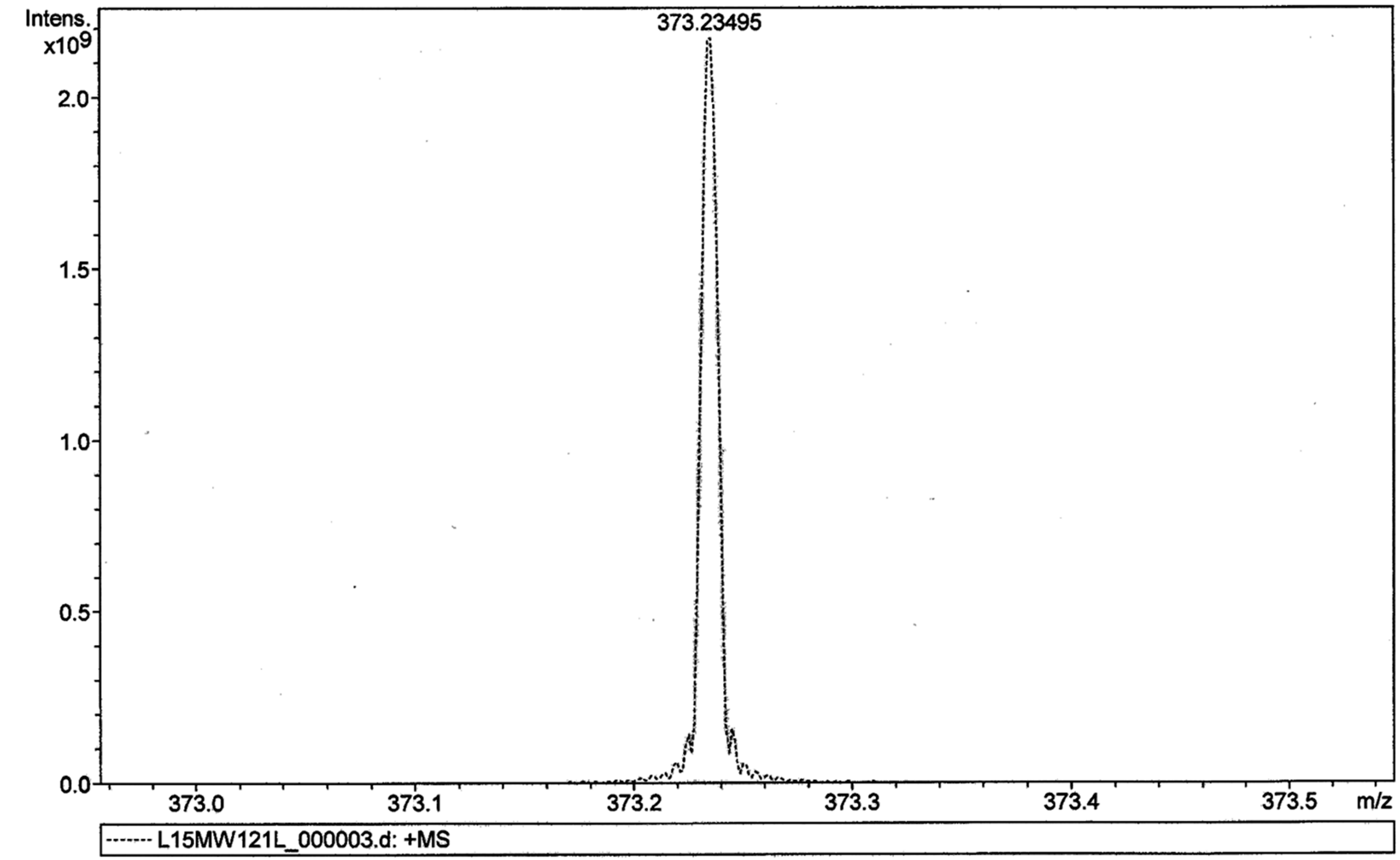

Meas. m/z	#	Formula	Score	m/z	err [mDa]	err [ppm]	mSigma	rdb	e⁻ Conf	N-Rule
373.23495	1	C 21 H 34 Na O 4	100.00	373.23493	-0.02	-0.06	4.8	4.5	even	ok

Figure S4. HRESIMS spectrum of **2**

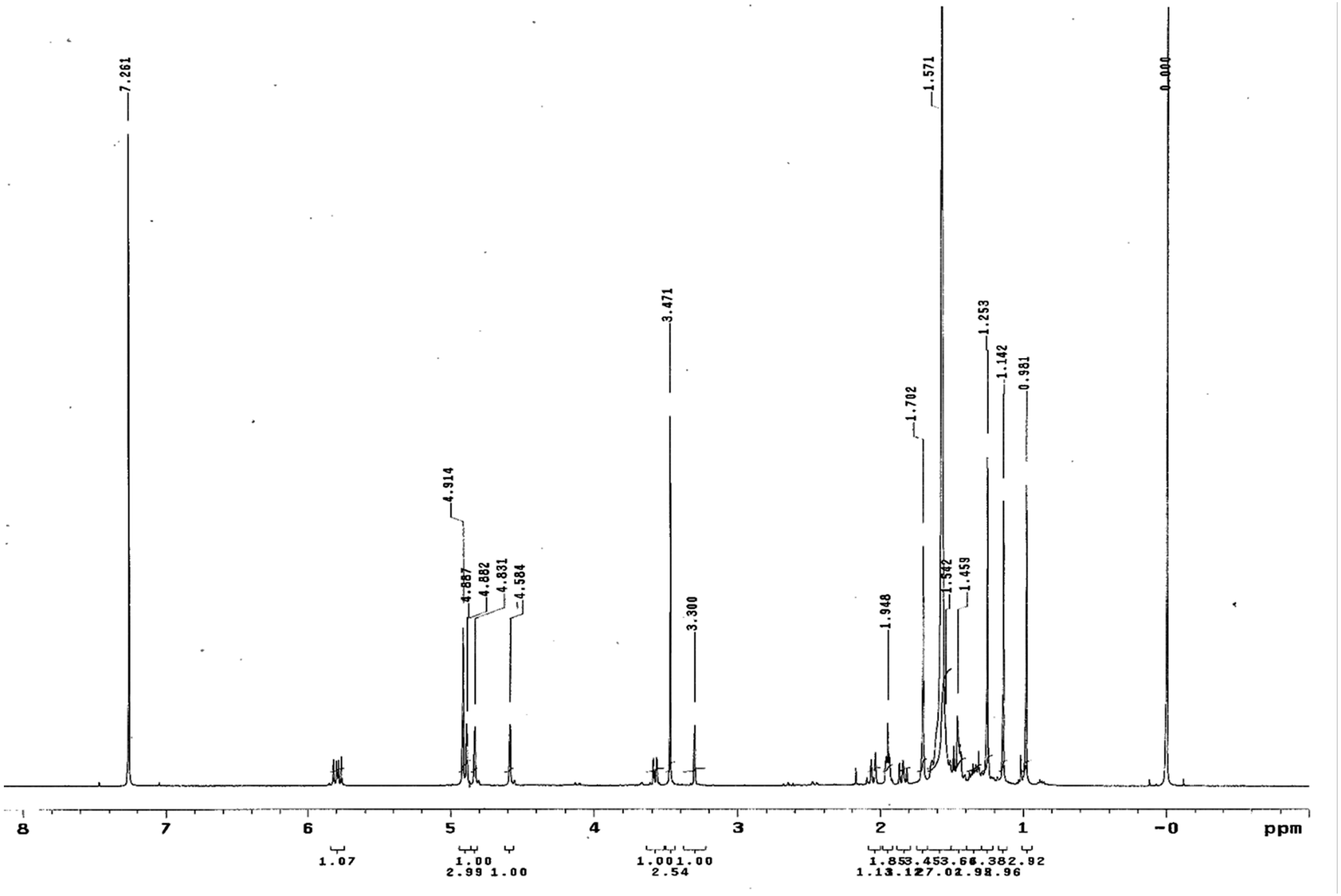

Figure S5. ^{1}H NMR spectrum of **2** in $CDCl_3$ at 500 MHz

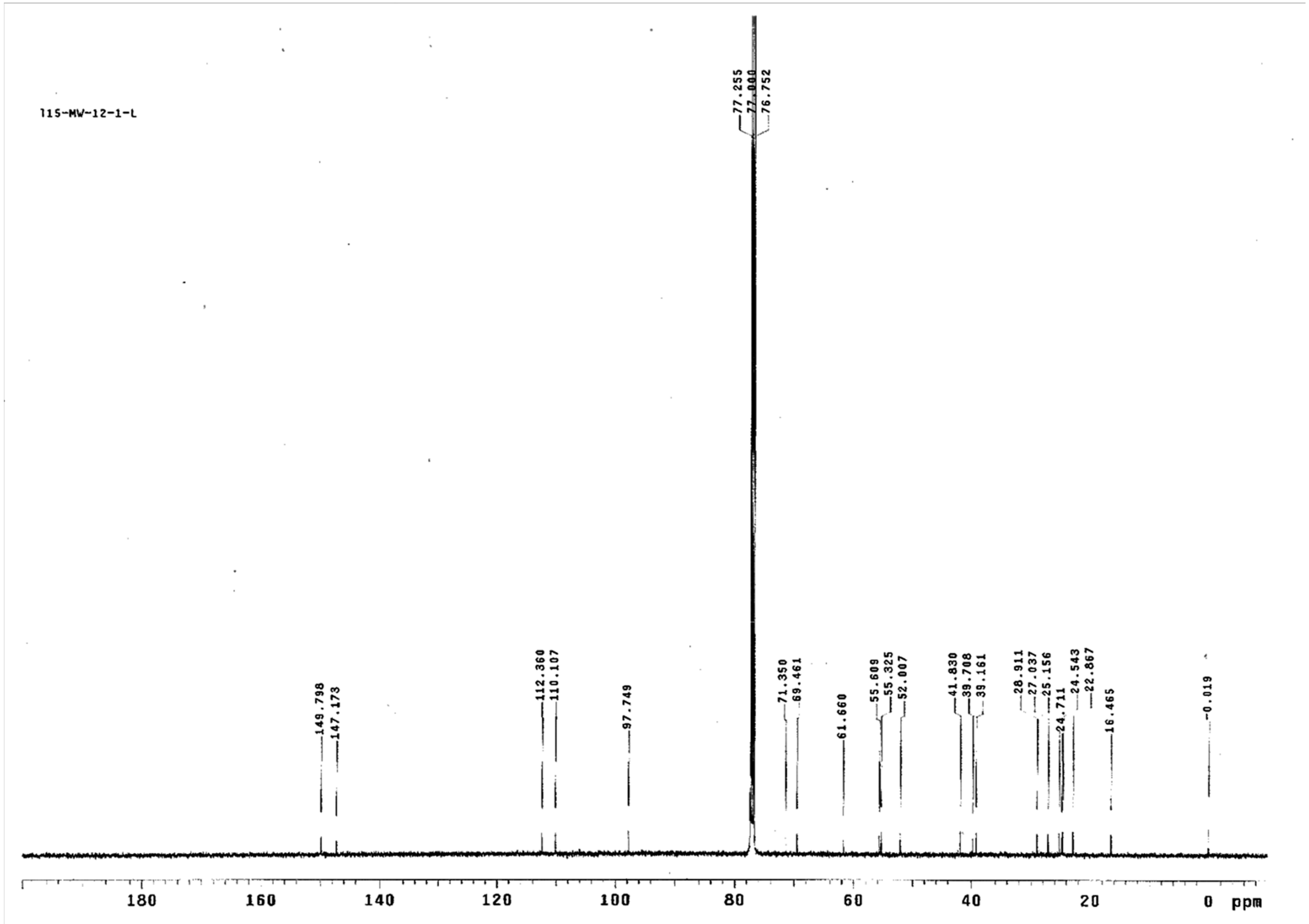

Figure S6. ^{13}C NMR spectrum of **2** in $CDCl_3$ at 125 MHz

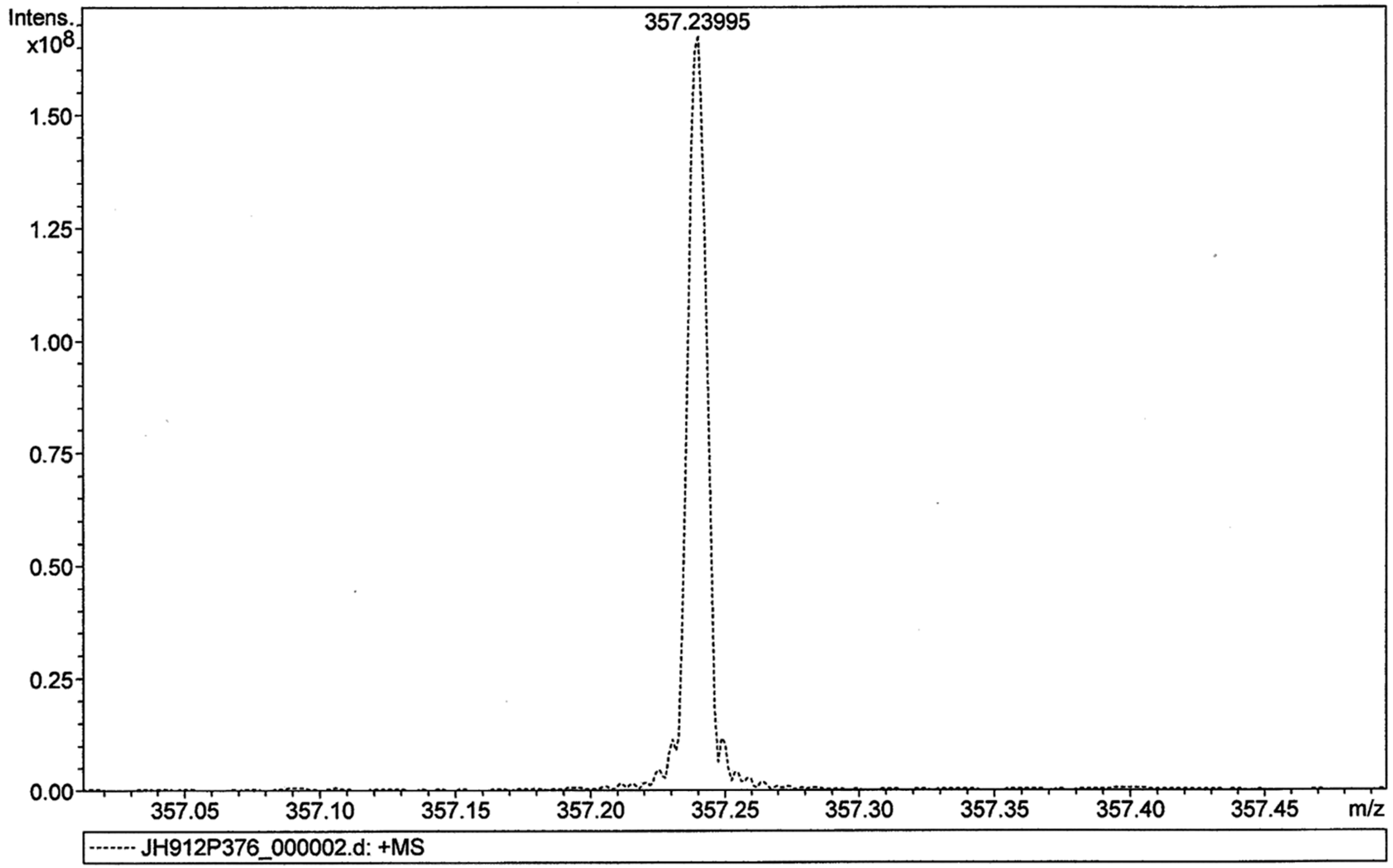

Meas. m/z	#	Formula	Score	m/z	err [mDa]	err [ppm]	mSigma	rdb	e⁻ Conf	N-Rule
357.23995	1	C 21 H 34 Na O 3	100.00	357.24002	0.06	0.18	6.5	4.5	even	ok

Figure S7. HRESIMS spectrum of **3**

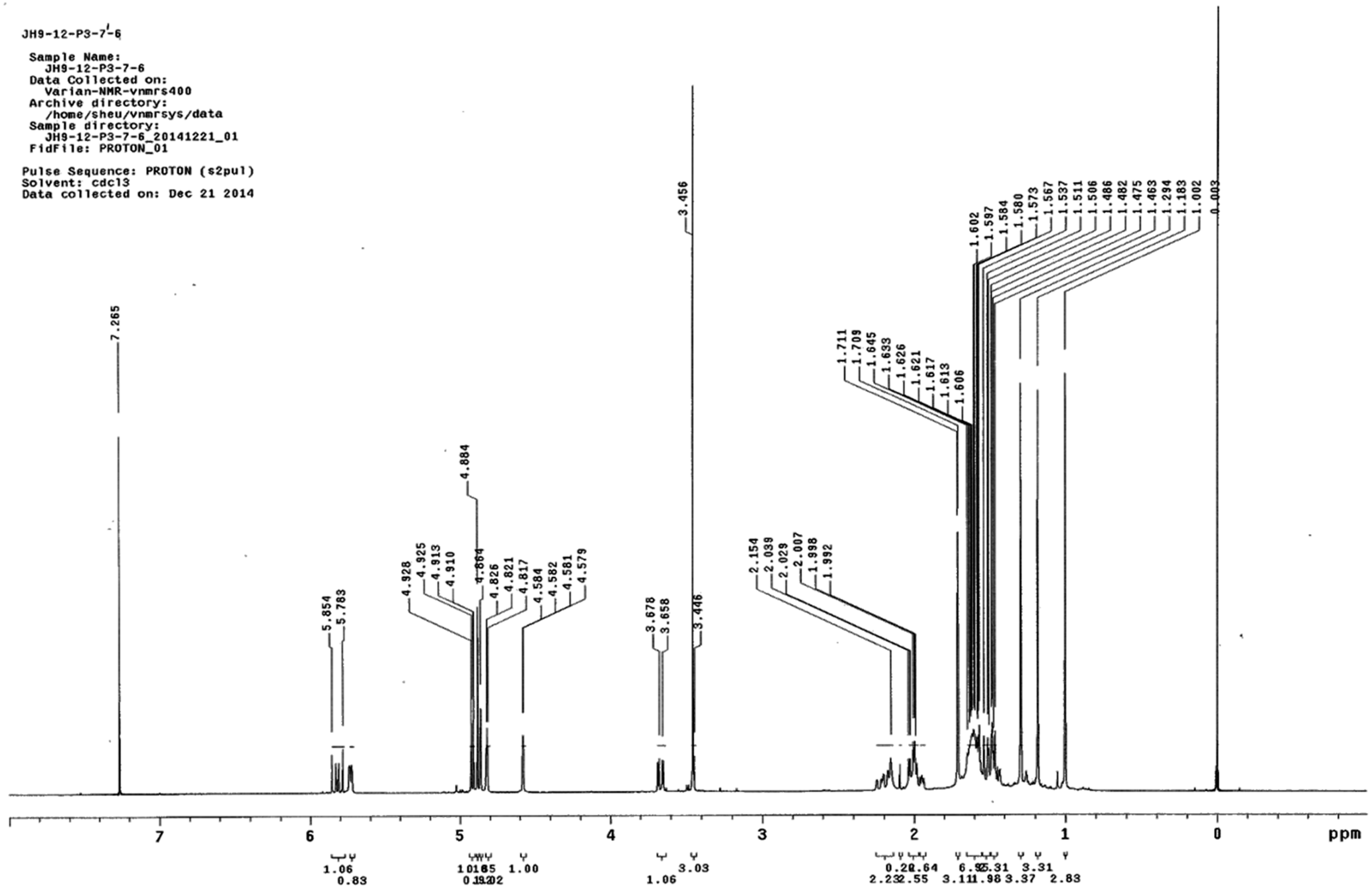

Figure S8. ¹H NMR spectrum of **3** in $CDCl_3$ at 400 MHz

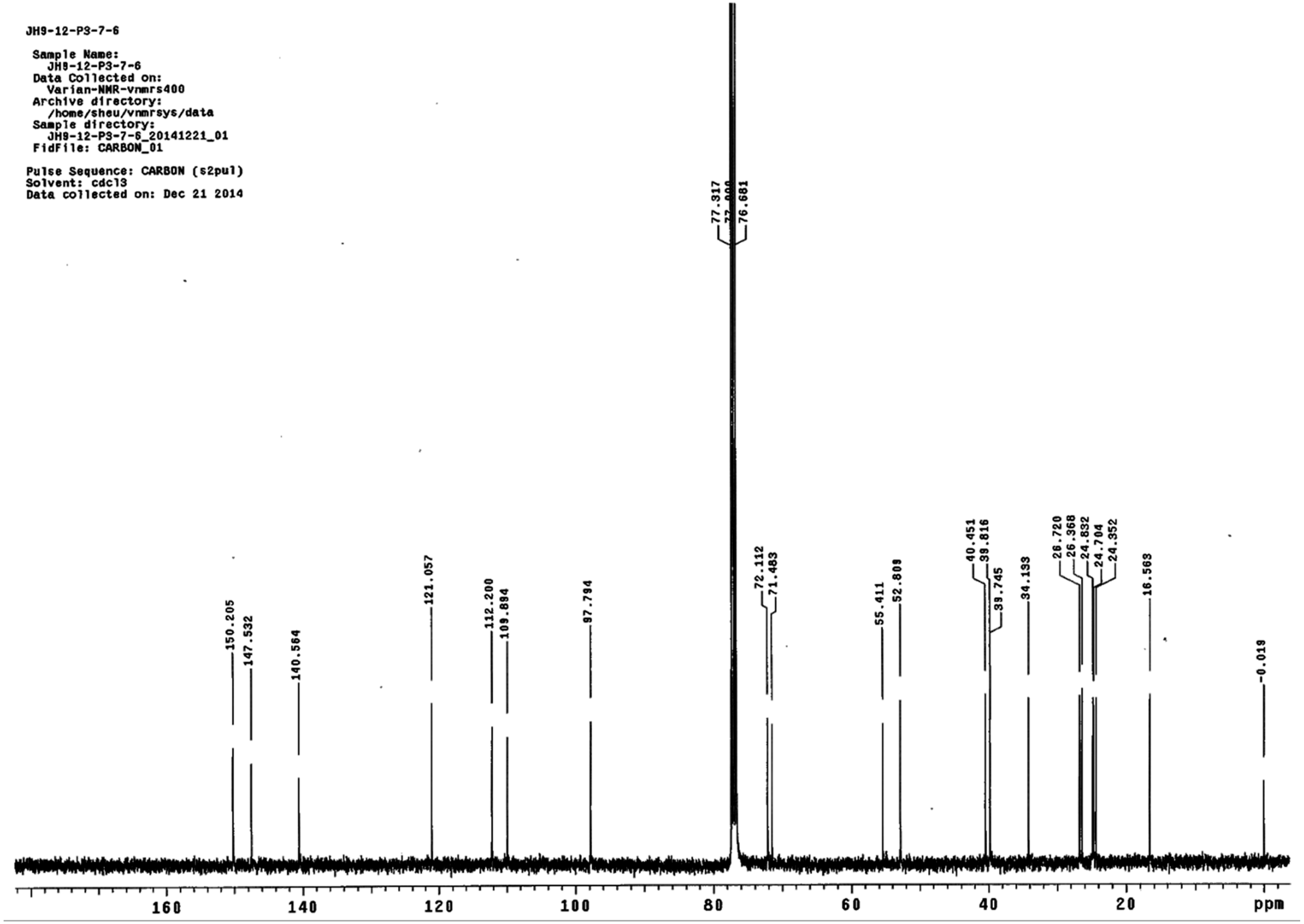

Figure S9. ^{13}C NMR spectrum of **3** in $CDCl_3$ at 100 MHz

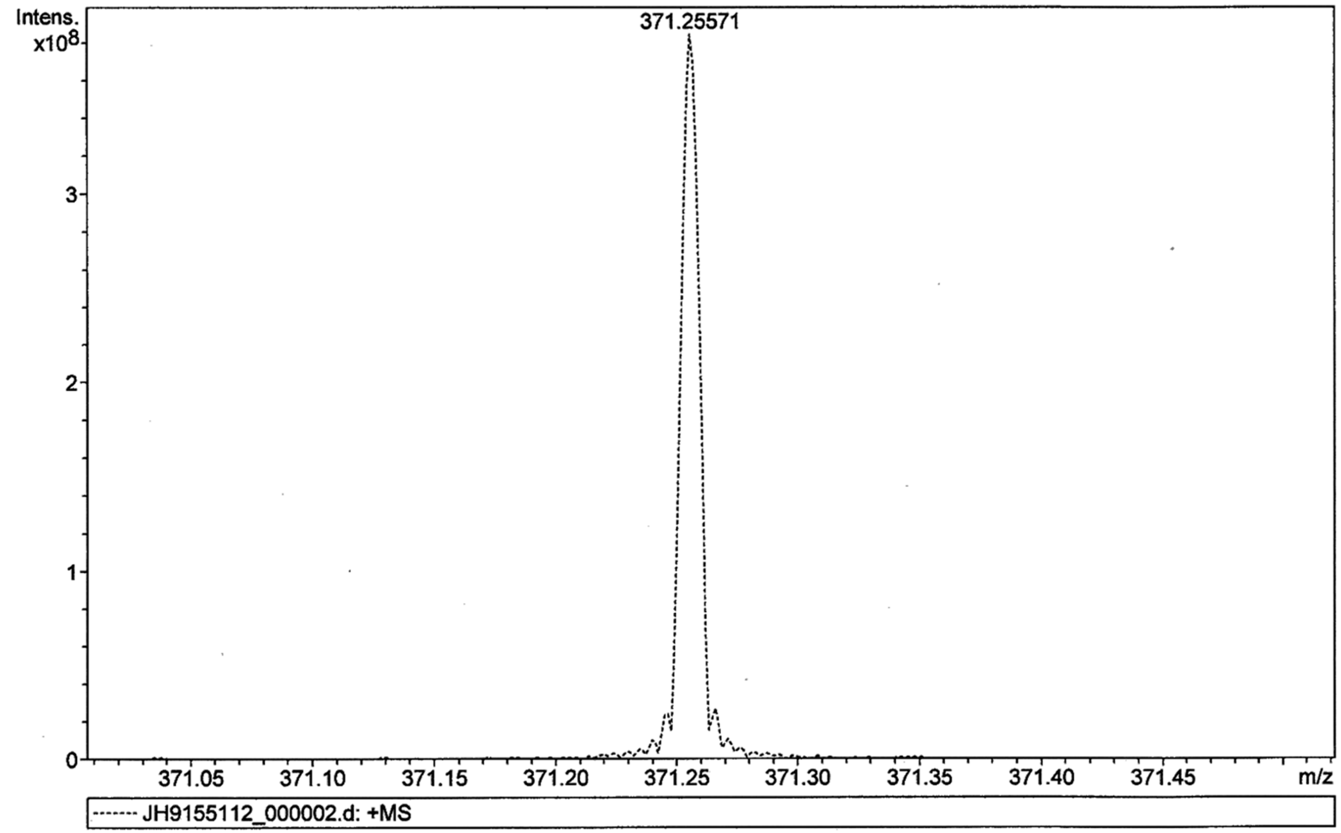

Meas. m/z	#	Formula	Score	m/z	err [mDa]	err [ppm]	mSigma	rdb	e⁻ Conf	N-Rule
371.25571	1	C 22 H 36 Na O 3	100.00	371.25567	-0.04	-0.11	3.0	4.5	even	ok

Figure S10. HRESIMS spectrum of **4**

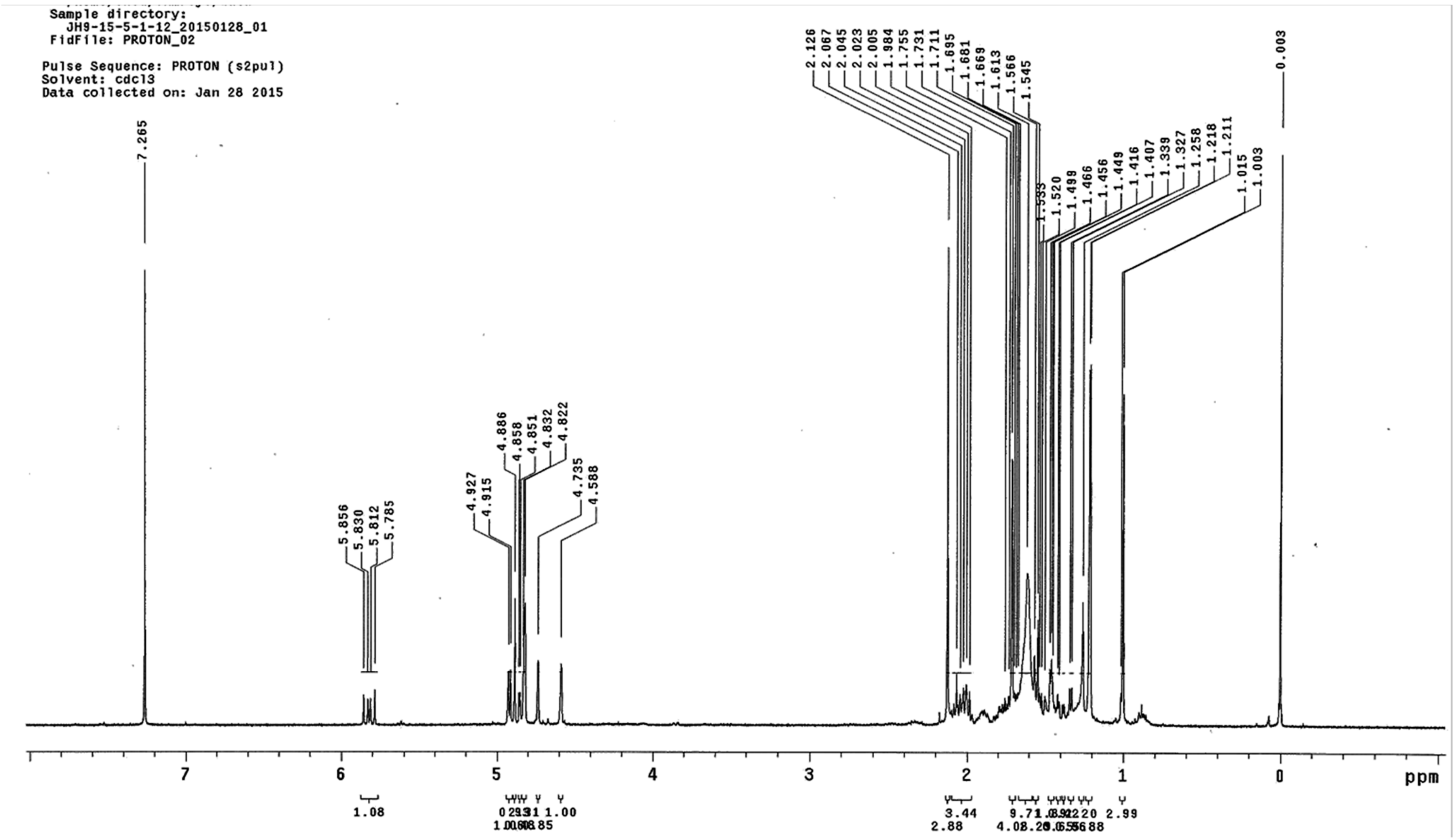

Figure S11. ^{1}H NMR spectrum of **4** in $CDCl_3$ at 400 MHz

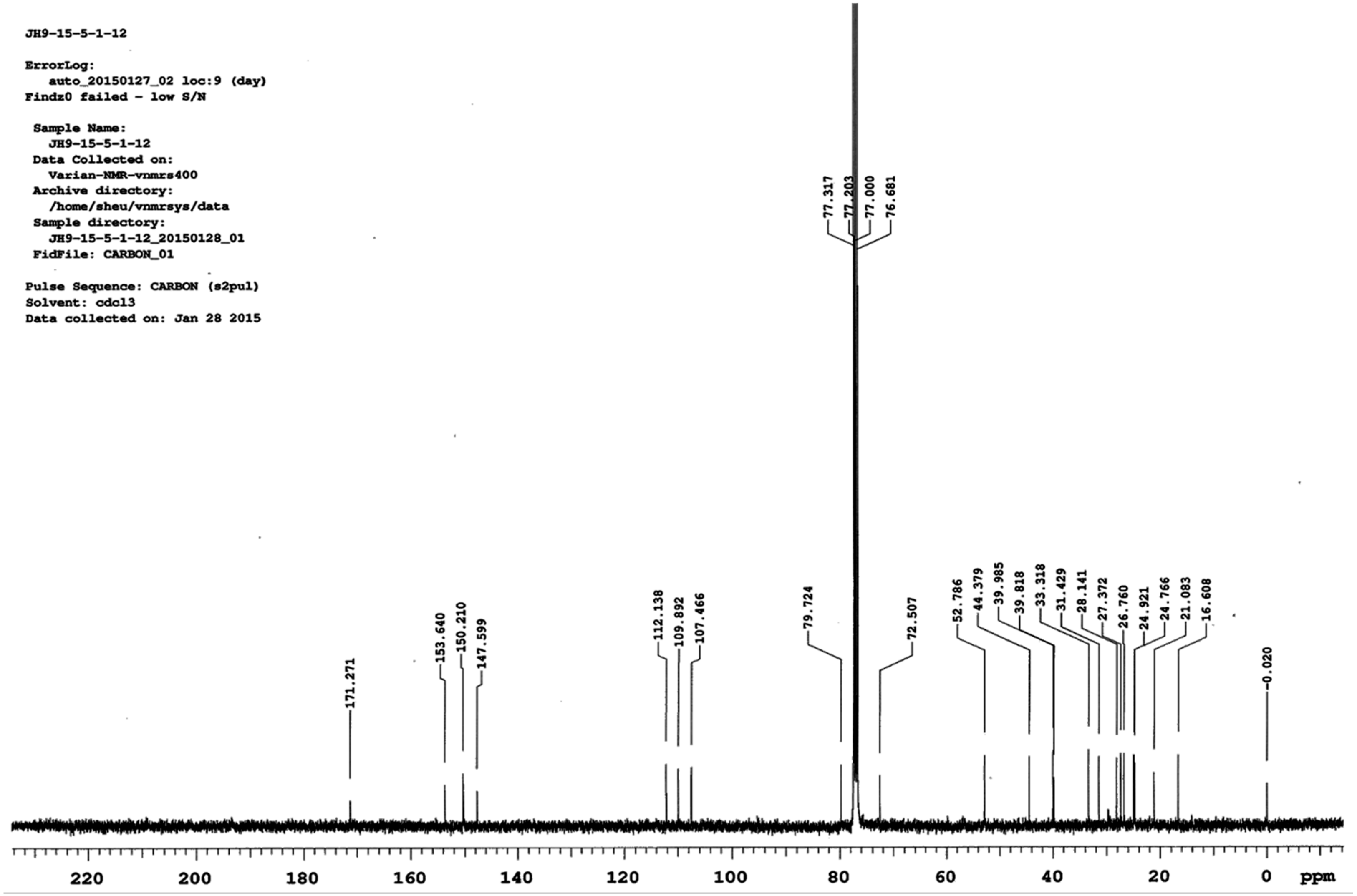

Figure S12. ^{13}C NMR spectrum of **4** in $CDCl_3$ at 100 MHz

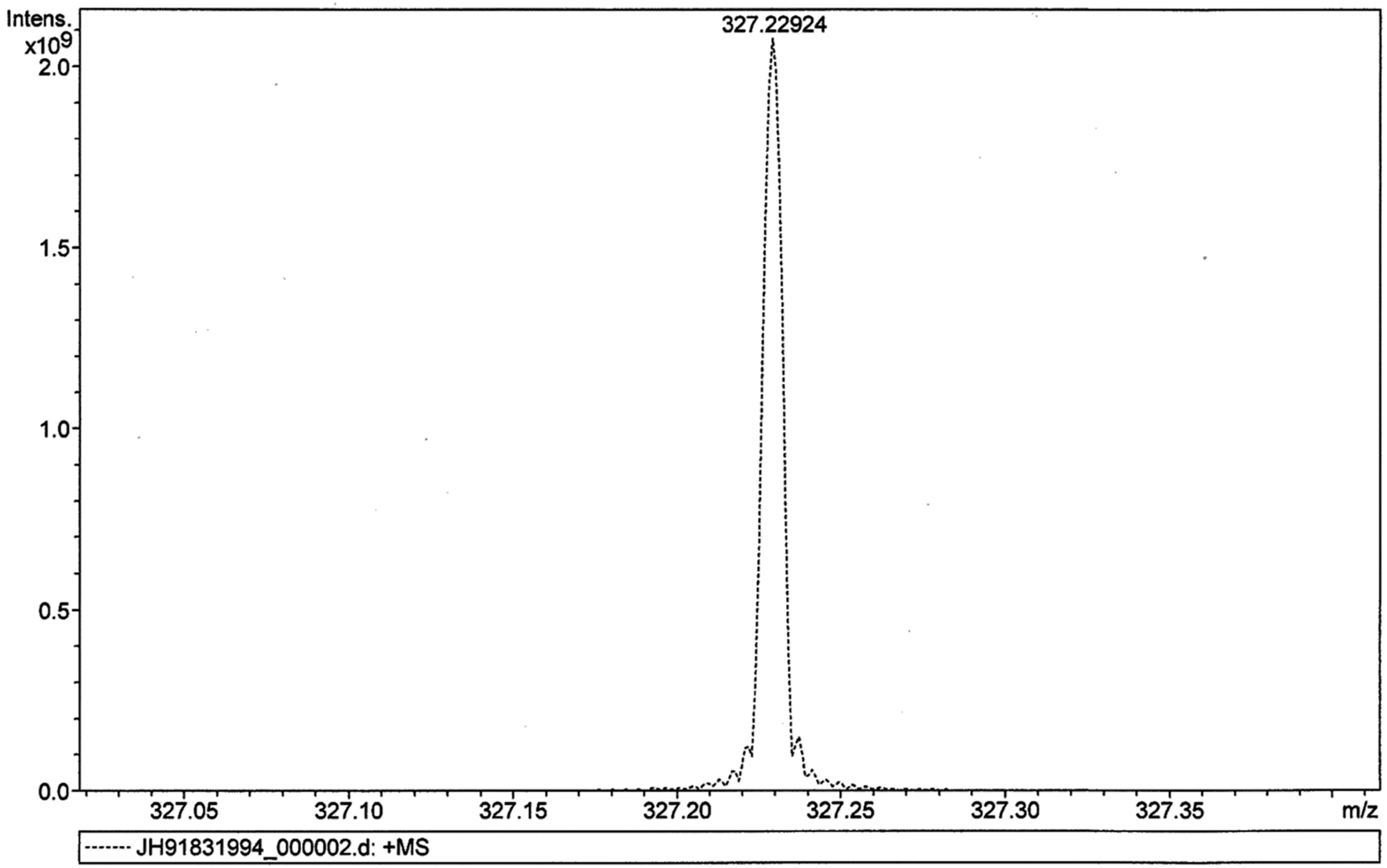

Meas. m/z	#	Formula	Score	m/z	err [mDa]	err [ppm]	mSigma	rdb	e⁻ Conf	N-Rule
327.22924	1	C 20 H 32 Na O 2	100.00	327.22945	0.21	0.65	5.6	4.5	even	ok

Figure S13. HRESIMS spectrum of **5**

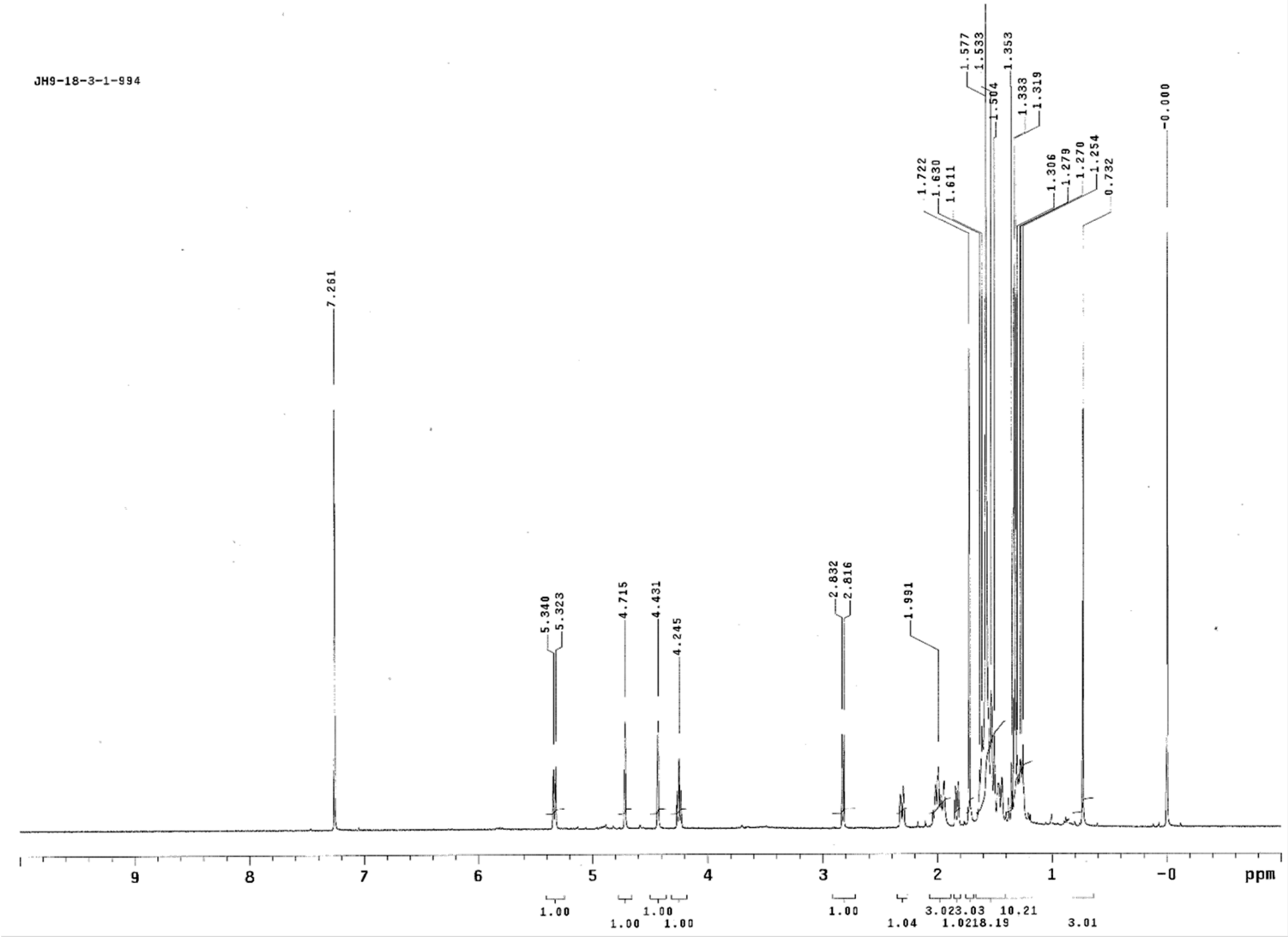

Figure S14. ^{1}H NMR spectrum of **5** in $CDCl_3$ at 500 MHz

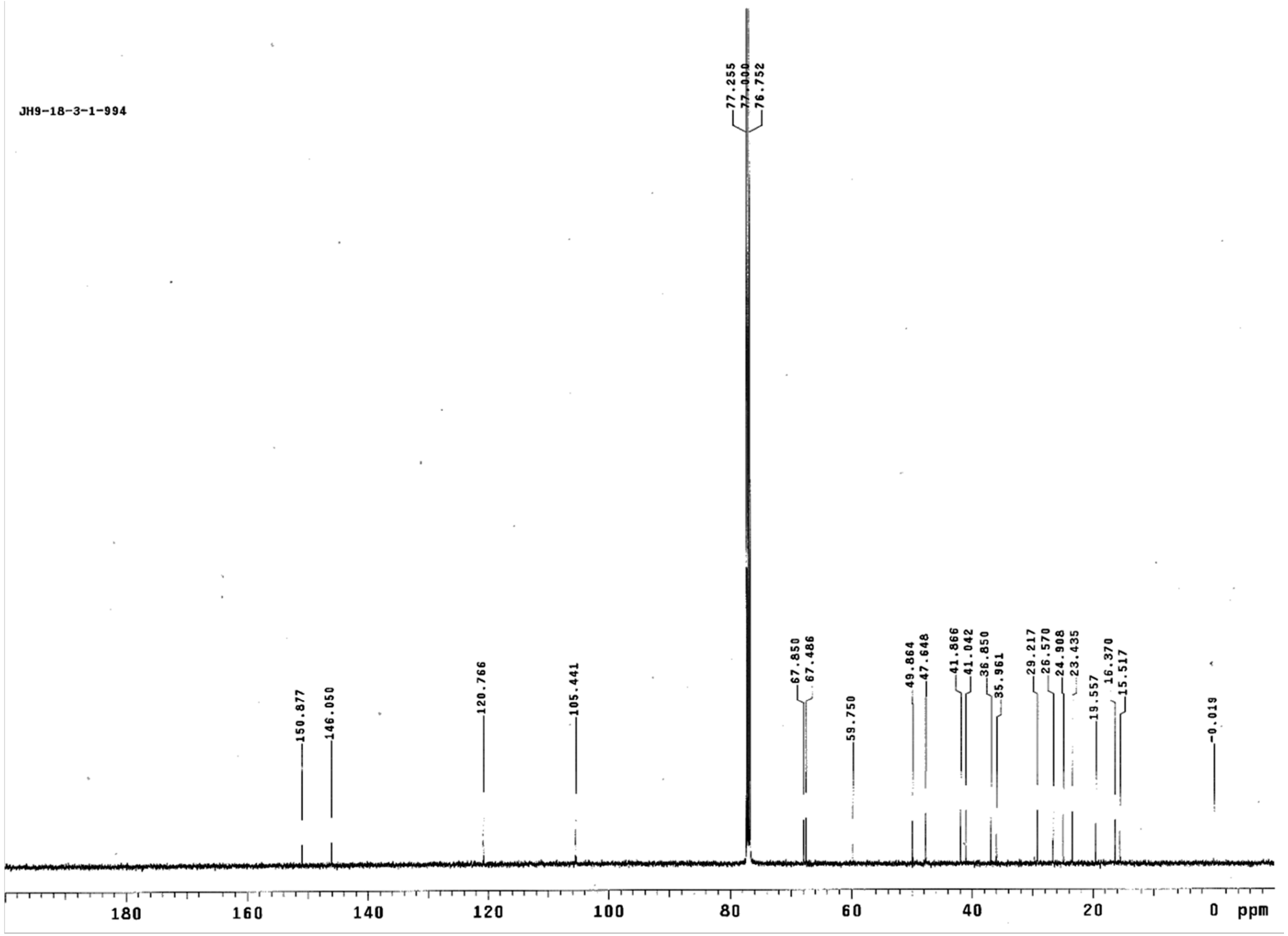

Figure S15. ^{13}C NMR spectrum of **5** in $CDCl_3$ at 125 MHz
